# The High Ratio of the Plasma miR-96/miR-99b Correlated With Poor Prognosis in Patients With Metastatic Colorectal Cancer

**DOI:** 10.3389/fmolb.2021.799060

**Published:** 2022-01-03

**Authors:** Yi Chen, Haizhou Liu, Shufang Ning, Changhong Wei, Jilin Li, Wene Wei, Litu Zhang

**Affiliations:** ^1^ Department of Research, Guangxi Medical University Cancer Hospital, Nanning, China; ^2^ Guangxi Cancer Molecular Medicine Engineering Research Center, Nanning, China

**Keywords:** miRNAs, colorectal cancer, metastasis, diagnosis, prognosis

## Abstract

**Object:** This study aims to clarify the expression of plasma miRNA in CRC patients, and to clarify the potential use of these miRNAs in diagnosis and prognosis, and to establish a prognostic model to initially explore its clinical value.

**Methods:** We detected the expression of 6 miRNAs in normal colon epithelial cell lines and colorectal cancer cell lines by qRT-PCR and they were validated in the tissues of three subtypes: 20 healthy subjects, 41 pCRC and 49 mCRC patients. COX regression and ROC analyses use to evaluate the diagnostic and prognostic efficacy of candidate miRNAs. Subsequently, we initially established a nomogram prognostic model. MiRNA is also used to construct miRNA-mRNA interaction network and PPI network modules.

**Results:** Five miRNAs showed significant differential expression in pCRC, mCRC patients and normal groups. ROC analysis showed that CEA, miR-96, miR-99b and miR-96/miR-99b are distinguishable from pCRC and mCRC patients, with AUC ranging from 0.65 to 0.91; among them, the ratio of miR-96/miR-99b is stronger than any diagnostic indicators, such as CEA and CA125. Multivariate survival analysis identified miR-96, miR-99b, N stage, M stage and clinical stage as independent prognostic indicators of mCRC. The nomogram based on these 5 characteristics has satisfactory prognostic values.

**Conclusion:** Our data indicate that plasma miR-96/miR-99b can be used as a promising biomarker for early detection of mCRC patients; our nomogram has a promising evaluation value.

## Introduction

Colorectal cancer (CRC) is one of the leading causes of cancer-related deaths (Sung et al., 2021). The occurrence of colorectal liver metastasis (mCRC) is a multi-factor and multi-step process. About 20–25% of CRC patients are diagnosed with liver metastasis initially, and up to 30–50% of patients with liver metastases after primary tumor resection (Hur et al., 2017). Most patients with mCRC are not suited for radical resection surgery with its characteristic low five-year-survival rate ≤10% (Manfredi et al., 2006). Common strategies, such as laparoscopy, fine needle aspiration cytology and imaging modalities, such as enhanced computed tomography (CT), magnetic resonance imaging (MRI), are either invasive, characterized by a high degree of complication or the insufficiency of typical symptoms or signs (Metcalfe et al., 2004). Due to the limitations of CEA, we urgently require more effective biomarkers for mCRC.

MiRNAs consist of short (18–25 nucleotides), endogenous, non-coding RNAs, which regulate the expression of target genes at the post-transcriptional level through base-repair binding to their3″ untranslated regions (UTRs), thus, enhancing the inhibition of translation or messenger RNA cleavage (Bartel, 2009). Up to 20–30% of gene expression regulate by miRNAs (Kent and Mendell, 2006). Alteration and dysfunction of miRNAs play essential roles during carcinogenesis, metastasis, and recurrence by regulating biological and pathological processes (Yang et al., 2013). Many studies have shown that some miRNAs may be prognostic markers and therapeutic targets for CRC, including (Qin et al., 2017). A recent study showed that a circulating-miRNA panel can improve the risk prediction ability of miRNAs (Zhang et al., 2018). MiR-211 and miR-25 in the blood are used as diagnostic indicators for CRC (Gu et al., 2018). Another study also showed that Mir-208b in exosomes CRC patients could be used as an indicator of oxaliplatin response (Raut et al., 2021). In subsequent studies, miRNA-1290 in serum has also shown to be potential predictors for CRC diagnosis and prognosis (Radwan et al., 2021). In plasma, an 8-miRNA-set including miR-17 (Ning et al., 2021) could distinguish colorectal adenoma from healthy control, and miR-141 can be used to predict stage IV colorectal cancer (Imaoka et al., 2016).

In recent years, some studies have verified metastatic miRNAs in tumor tissues (Kanaan et al., 2013). It demonstrates that some miRNAs have tissue and disease specificity. Therefore, whether miRNAs signatures in plasma can use as diagnostic markers for mCRC remains worthy of study.

**FIGURE 1:**
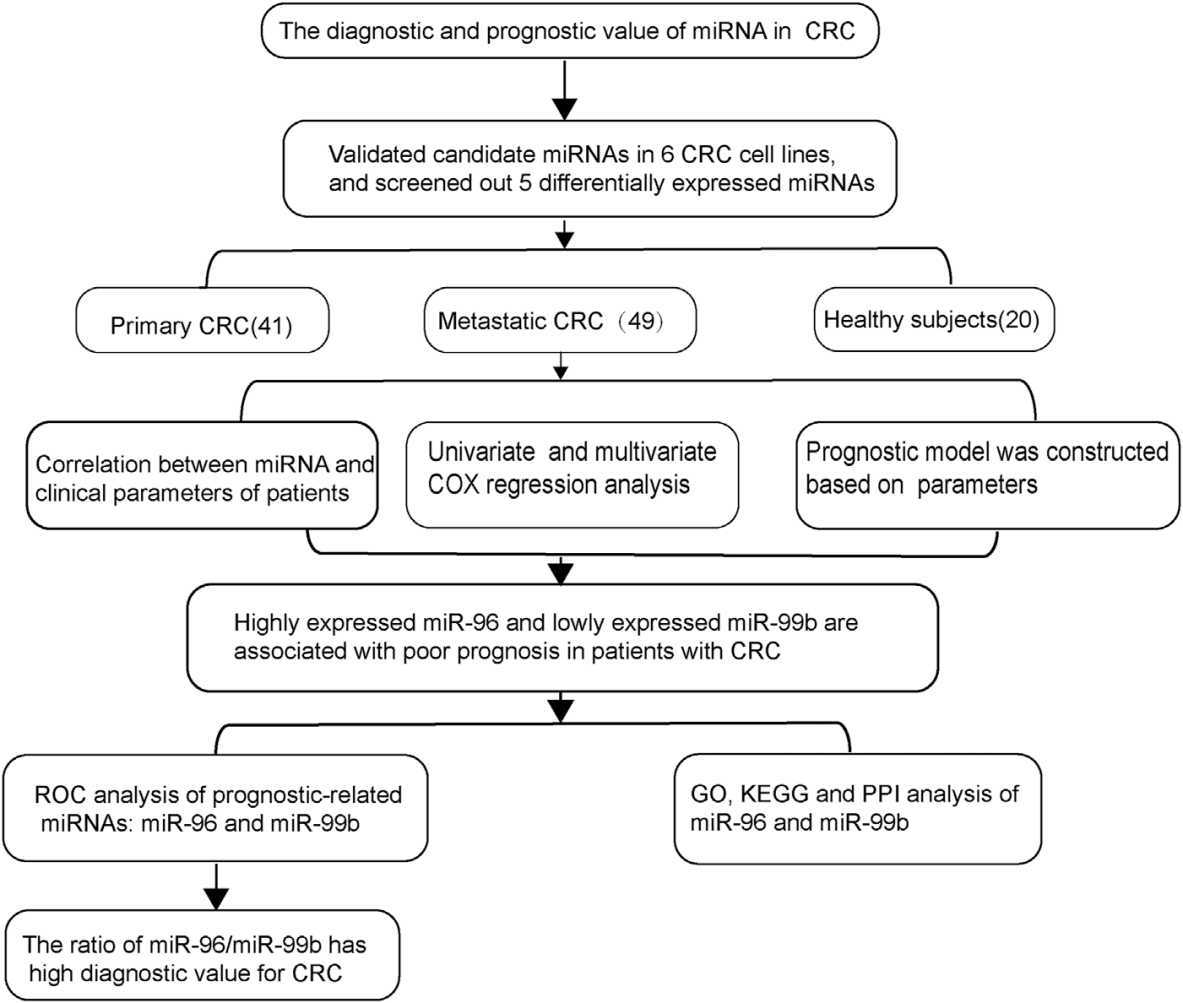
The workflow of the entire study.

Our recent work and other studies have suggested that six different miRNAs (miR-96, miR-99a, miR-99b, miR-155, let-7a (let-7a), and let-7b) are associated with tumor progression and distant metastasis (Cheng et al., 2011). Here we investigated the role of miRNAs in colorectal cancer cell lines and pCRC and mCRC patients. We found that five differentially expressed miRNAs were associated with mCRC. Subsequently, we evaluated the diagnostic and prognostic values of these five candidate miRNAs for mCRC (Figure 1).

## Materials and Methods

### Acquisition of miRNA Expression Data

We screened the differentially expressed miRNAs in mCRC patients through the GEO database.

### Cell Culture

Human colorectal cancer cell line (HCT16, LoVo, SW480, SW620, DLD1, RKO) and normal colon epithelial cell line NCM460 purchase from Shanghai Institute of Biological Sciences, Chinese Academy of Sciences. CRC cell lines were all cultured in Dulbecco’s modified Eagle’s medium (DMEM), supplemented with 10% FBS (both Gibco; Thermo Fisher Scientific, Inc.), in a humidified atmosphere at 5% CO2 and 37°C.

### Patient’s Selection and Sample Collection

A total of 110 subjects enrolled from Guangxi Medical University Cancer Hospital between December 2009 and December 2017. The Clinical Research Ethics Committee approved this study of Guangxi Medical University Cancer Hospital. Each subject had provided written informed consent for the use of specimens and clinical data. Excluding co-existing diseases, plasma samples obtain from patients with CRC (stage I ∼ IV) before colorectal surgical treatment, preoperative radiotherapy or chemotherapy, which was consist of two subtypes, 41 stage I-III non-metastasis CRC patients and 49 stage IV mCRC patients. All stage I-III patients were diagnosed with no distant metastases and stage IV patients had liver metastases. Pathological classification of all patients was performed according to the International Union Against Cancer (UICC) and the American Joint Committee on Cancer (AJCC) for colon cancer. In the control group, 20 blood samples collected from age- and gender-matched healthy subjects who confirm to be without any malignancy or another benign disease after physical examination. Plasma samples from patients with different levels of CEA were acquired from the clinical database of Guangxi Medical University Cancer Hospital and measured by standard enzyme immunoassay. The overall survival (OS) data of all CRC patients were received from clinical databases, and the survival time is from the date of diagnosis to the date of cancer-related death or the date of the last follow-up. The median follow-up time was 87.8 months.

The blood samples were processed in tubes containing EDTA. Separate cell-free plasma from blood samples within 4 h of collection using a two-step protocol (1,900 × *g* for 10 min, 16,000 × *g* for 10 min) and then stored at −80°C until further processing.

### RNA Extraction

RNA was extracted using the miRNeasy Serum/Plasma Kit (Cat no 217184, Qiagen). Each piece eluted in 14 μL of RNase-free water using Eppendorf Concentrator Plus 5301 (Eppendorf, Germany). The NanoDrop 2000 spectrophotometer used to measure the concentration and purification of total RNA. RNA stored at −80°C for subsequent qPCR assays.

### Quantitative Real-Time PCR

The same amount of *Caenorhabditis* Elegans cel-39-3p miRNA spiked into each plasma sample as an external calibration for RNA extraction, reverse transcription, and miRNA amplification. Total RNA was reverse transcribed into cDNA with the miScript II RT kit (Qiagen, Hilden, Germany) following the manufacturer’s instructions. The products of R.T. were analyzed using an Agilent Mx3000 qPCR system with the miScript SYBR-Green PCR kit (Qiagen) under the manufacturer’s instructions. Each sample analyzed in triplicate. The primer sequences are as follows: UAG​CAG​CAC​GUA​AAU​AUU​GGC​G for hsa-miR-16-5p; UUU​GGC​ACU​AGC​ACA​UUU​UUG​CU for hsa-miR-96-5p; AAC​CCG​UAG​AUC​CGA​UCU​UGU​G for hsa-miR-99a-5p; CAC​CCG​UAG​AAC​CGA​CCU​UGC​G for hsa-miR-99b-5p; UUA​AUG​CUA​AUC​GUG​AUA​GGG​GU for hsa-miR-155-5p; UGA​GGU​AGU​AGG​UUG​UAU​AGU​U for hsa-let-7a-5p; UGA​GGU​AGU​AGG​UUG​UGU​GGU​U for hsa-let-7b-5p. MiR-16 was used as the housekeeping gene due to its previously published literature (O'Connor et al., 2013), and its usability and effectiveness have verify in our study (Supplementary Table S1). The qPCR primers come from Tiangen Biotech (Beijing) Co. Ltd. All PCR reactions were performed on an Agilent Mx3000 System and the relative levels of miRNAs expression calculated by the 2^−ΔΔCt^ method.

### Construction and Verification of Prognostic Model

First, Cox regression was used to screen independent risk factors related to prognosis. The independent prognostic factors obtained were used as independent variables to draw the Nomogram model to evaluate the prognosis of CRC patients. Next, we evaluate the discrimination of the prediction model constructed in the above two steps and calculate the C-Index. Finally, we use the Bootstrap resampling method to perform internal verification based on the data set and draw a calibration curve (Wang et al., 2013).

### Prediction of MiRNAs Target Genes

The prediction of target candidates of selected miRNAs was performed using MiRWalk3.0 (http://mirwalk.umm.uni-heidelberg.de/). A functional enrichment analysis was conducted on all predicted target genes with miRWalk 3.0 (the newest version).

### Functional Enrichment Analysis

To clearly elucidate the function and regulatory model of miRNAs at the cellular level, analyses including, G.O. and KEGG analyses of the miRNAs were performed using the clusterProfiler package (R software package, version 2.4.3). Result with *p* < 0.05 and count >2 is of significance. DIANA-miR Path (http://diana.cslab.ece.ntua.gr/pathways/) was adopted to perform online gene enrichment analysis of putative targets. Subsequently, KEGG was achieved to analyze the function of target genes.

### PPI Network Construction and Module Analysis

Protein-protein interaction (PPI) network of target genes was construct by the Search Tool for the Retrieval of Interacting Genes (STRING, version 11.0, https://string-db.org/). STRING was a promising and user-friendly online database which may further illustrate the mechanisms between interactive genes. By applying STRING database, PPI network of genes was conducted and an interaction with a combined score >0.4 was defined as statistical significance. The plug-in Molecular Complex Detection (MCODE) app of Cytoscape (version 3.7.1) is constructed for clustering a interaction network to identify intensively correlated regions (Srihari and Leong, 2012). The PPI network was visualized with the app of Cytoscape and the most crucial module in PPI network was obtained using MCODE with the following criteria: node score cutoff = 0.2, degree cutoff = 2, k-core = 2 and max depth = 100.

### Statistical Analysis

The expression levels of selected miRNAs were presented as mean ± standard deviation (mean ± S.D.) Student’s *t*-test, one-way ANOVA or non-parametric tests were used to assess differences in miRNA levels between different groups. Receiver operator characteristic (ROC) analysis was constructed to obtain diagnostic utility of miRNAs. Binary logistic regression analysis was used to access the diagnostic performance of the combination panels of miRNAs. Univariate and multivariate Cox proportional hazard models were performed. Survival curves were compared with log-rank test. All independent risk factors were analyzed in R software and rms packages were applied to establish nomogram model. Bootstrap analysis was conducted to perform internal validation. *p* < 0.05 is considered statistically significant.

## Results

### MiRNAs Expression Data Mining

Differentially expressed miRNAs in primary/metastatic colorectal cancer tissues. The selected dataset is GSE72199 (Table 1). Chip samples collected from 28 primary colorectal cancer and eight liver metastases. According to whether there was statistical significance and the difference multiple, the qualified differential miRNA expression was shown in Supplementary Table S2.

**TABLE 1:** Chip set of metastatic colorectal cancer.

GEO	Subject	Type	Included
GSE72199	Human colon cancer tissues and liver metastases	GPL15018	GSM 1857440 GSM1857441 GSM1857442 GSM1857443 GSM1857444 GSM1857445 GSM1857446 GSM1857447 GSM 185 7448 GSM185 7449 GSM1857450 GSM1857451 GSM1857452 GSM1857453 GSM1857454 GSM1857455 GSM 1857456 GSM1857457 GSM1857458 GSM1857459 GSM1857460 GSM1857461 GSM1857462 GSM1857463 GSM 1857464 GSM1857465 GSM1857466 GSM1857467 GSM1857472 GSM1857473 GSM1857474 GSM1857475 GSM 1857468 GSM1857469 GSM1857470 GSM1857471

In order to comprehensively evaluate the diagnostic and prognostic value of the above differential miRNAs for CRC/liver metastasis, six miRNAs including miR-96, miR-99a/b, miR-155, let-7a and let-7b were selected for subsequent studies according to the inclusion and exclusion criteria. The differences of miRNAs involved in the literature are shown in Supplementary Table S3.

### MiRNAs in Cell Lines

**FIGURE 2:**
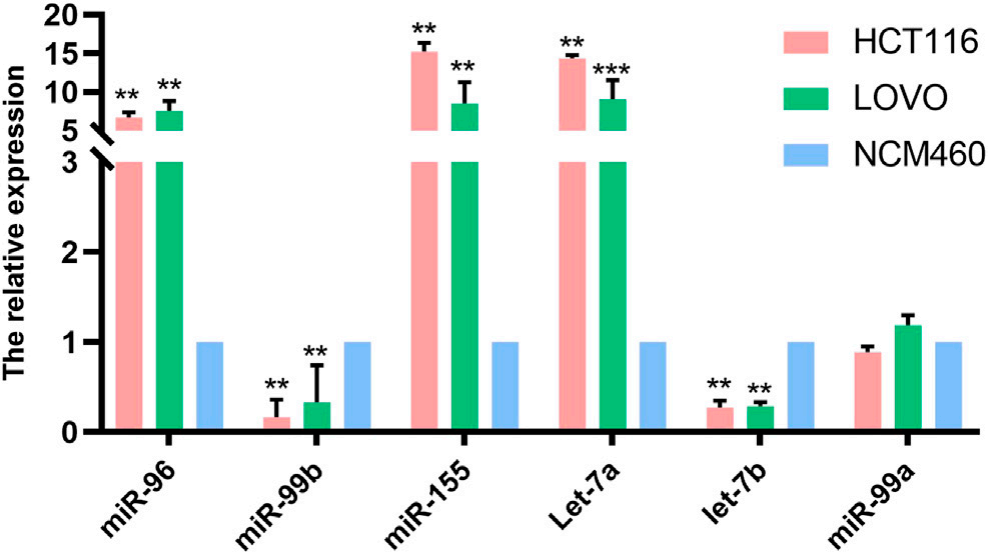
The expression of miRNAs in normal intestinal epithelial NCM460 cell lines, HCT116 and LoVo were detected by qPCR. * Means *p* < 0.05, and ** means *p* < 0.01.

We examined the expression of six miRNAs in intestinal epithelial cell lines and intestinal cancer cell lines. Our study found that, compared with NCM460 cells, miR-96, miR-99b, miR-155, let-7a and let-7b were differentially expressed in HCT116 and LoVo cell lines, while no significant differences were found in the expression of miR-99a. Among them, the level of miR-96, miR-155 and let-7a were up-regulated in HCT116 and LoVo, while miR-99b and let-7b were down-regulated (Figure 2).

### Study Population

The study consisted of 90 CRC patients and 20 healthy controls. No significant differences were detected between the CRC patients and authorities in the distribution of gender and age. Clinico-pathological characteristics of all subjects summarize in Table 2.

**TABLE 2:** Baseline characteristics of 90 CRC patients and healthy controls.

Characteristics	Patients	Control	*p* value
Gender	—	—	—
Male	55	12	0.17^a^
Female	35	8	—
Age	—	—	0.08^a^
<60	50	11	—
≥60	40	9	—
Degree of differentiation	—	—	—
Poor	25	—	—
Well	65	—	—
Tumor diameter(cm)	—	—	—
<5	54	—	—
≥5	36	—	—
T stage	—	—	—
Tis-T2	11	—	—
T3-T4	79	—	—
N stage	—	—	—
Absent	22	—	—
Present	68	—	—
M stage		—	—
M0	41	—	—
M1	49	—	—
Clinical stage	—	—	—
Early stage (I-II)	2	—	—
Advanced stage (III-IV)	70	—	—
CEA (ng/L)	—	—	—
<5	42	—	—
≥5	48	—	—

a Note: : independent-samples *t* test.

### Correlation of MiRNAs Expression Levels With Clinico-Pathological Characteristics

We selected five miRNAs differentially expressed in cell lines for further study. We analyzed the association between all five miRNAs expression and clinicopathological features (Table 3). Expression level of the five miRNAs were correlated with stage T, N, M, and clinical stage. Moreover, miR-96 and miR-99b were related to the CEA level.

**TABLE 3:** Association of miRNAs expression levels with clinico-pathological factors of colorectal cancer patients (presented as mean ± SD).

Variable	N	MiR-96	*p* value	MiR-99b	*p* value	MiR-155	*p* value	Let-7a	*p* value	Let-7b	*p* value
Gender	—	—	0.34	—	0.82	—	0.40	—	0.46	—	0.39
Male	55	4.88 ± 1.25	—	0.52 ± 0.27	—	2.71 ± 1.24	—	2.63 ± 1.03	—	0.44 ± 0.29	—
Female	35	5.16 ± 1.41	—	0.51 ± 0.34	—	2.92 ± 1.09	—	2.78 ± 0.86	—	0.51 ± 0.42	—
Age	—	—	0.39	—	0.24	—	0.92	—	0.62	—	0.83
<60	50	5.10 ± 1.36	—	0.49 ± 0.28	—	2.78 ± 1.11	—	2.73 ± 0.95	—	0.46 ± 0.33	—
≥60	40	4.85 ± 1.23	—	0.56 ± 0.32	—	2.807 ± 1.30	—	2.63 ± 0.99	—	0.48 ± 0.38	—
Degree differentiation	—	—	0.48	—	0.78	—	0.17	—	0.66	—	0.07
Poor	256	4.83 ± 1.19	—	0.51 ± 0.27	—	3.07 ± 1.25	—	2.61 ± 0.86	—	0.38 ± 0.22	—
Well	65	5.05 ± 1.35	—	0.53 ± 0.31	—	2.68 ± 1.16	—	2.71 ± 1.01	—	0.50 ± 0.38	—
Tumor diameter (cm)	—	—	0.22	—	0.31	—	0.15	—	0.50	—	0.76
<5	54	4.85 ± 1.17	—	0.55 ± 0.31	—	2.64 ± 1.19	—	2.63 ± 0.94	—	0.48 ± 0.35	—
≥5	36	5.20 ± 1.89	—	0.48 ± 0.27	—	3.01 ± 1.18	—	2.77 ± 1.01	—	0.45 ± 0.34	—
T stage	—	—	0.01	—	0.02		0.03	—	0.004	—	0.001
Tis-T2	11	3.99 ± 1.16	—	0.78 ± 0.36	—	1.81 ± 0.36	—	1.79 ± 0.89	—	0.79 ± 0.59	—
T3-T4	79	5.13 ± 1.27	—	0.49 ± 0.27	—	2.93 ± 1.11	—	2.81 ± 0.91	—	0.42 ± 0.27	—
N stage	—	—	0.00	—	0.00	—	0.00	—	0.001*	—	0.00
N0	22	3.89 ± 1.09	—	0.84 ± 0.29	—	1.69 ± 0.80	—	1.71 ± 0.83	—	0.81 ± 0.42	—
N1+N2	68	5.34 ± 1.17	—	0.42 ± 0.22	—	3.15 ± 1.07	—	2.99 ± 0.78	—	0.36 ± 0.24	—
M stage	—	—	0.00	—	0.00	—	0.00	—	0.001*	—	0.00
Absent	41	4.08 ± 1.11	—	0.76 ± 0.27	—	2.13 ± 0.93	—	2.08 ± 0.84	—	0.62 ± 0.38	—
Present	49	5.75 ± 0.92	—	0.32 ± 0.12	—	3.35 ± 1.09	—	3.19 ± 0.76	—	0.34 ± 0.25	—
Clinical stage	—	—	0.00	—	0.00	—	0.00	—	0.001*	—	0.00
Early (I-II)	20	3.71 ± 0.95	—	0.91 ± 0.21	—	1.53 ± 0.50	—	1.53 ± 0.58	—	0.85 ± 0.41	—
Advanced (III-IV)	70	5.35 ± 1.16	—	0.41 ± 0.22	—	3.15 ± 1.08	—	3.01 ± 0.78	—	0.36 ± 0.23	—
CEA	—	—	0.03	—	0.01	—	1.13	—	0.09	—	0.05
<5 ng\L	42	4.66 ± 1.27	—	0.60 ± 0.20	—	2.59 ± 1.15	—	2.51 ± 0.88	—	0.55 ± 0.36	—
≥5 ng\L	48	5.28 ± 1.29	—	0.45 ± 0.29	—	2.97 ± 1.21	—	2.84 ± 1.02	—	0.40 ± 0.32	—

Note: Independent-samples *t* test was conducted.

### Plasma miRNAs Differentially Expressed in pCRC and mCRC

**FIGURE 3:**
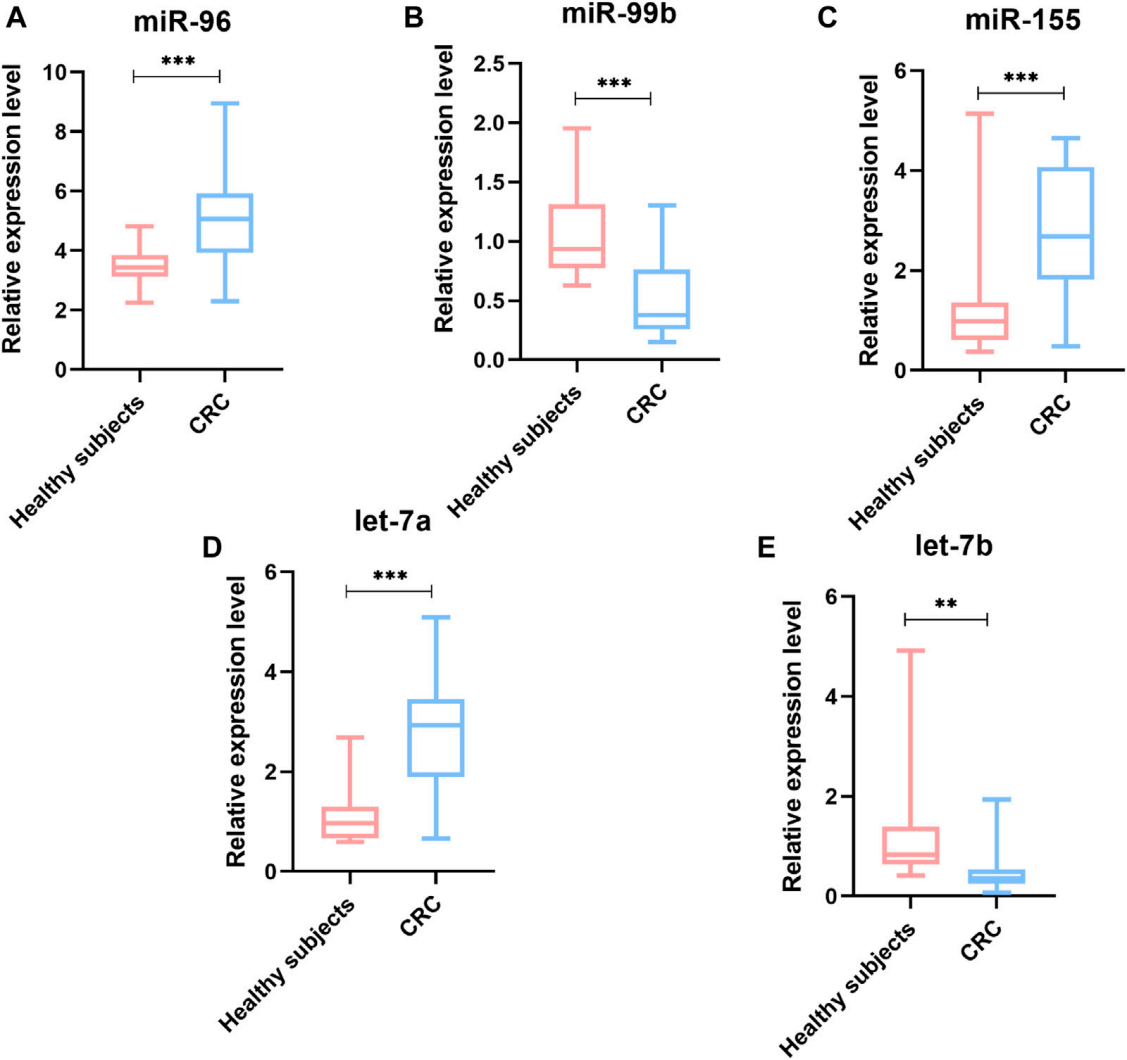
The relative expression level of miRNAs in plasma samples (90 CRC and 20 controls). **(A)** miR-96; **(B)** miR-99b; **(C)** miR-155; **(D)** Let-7a; **(E)** Let-7b; *p value < 0.05, **p value < 0.01 and *** means *p* value < 0.001.

**FIGURE 4:**
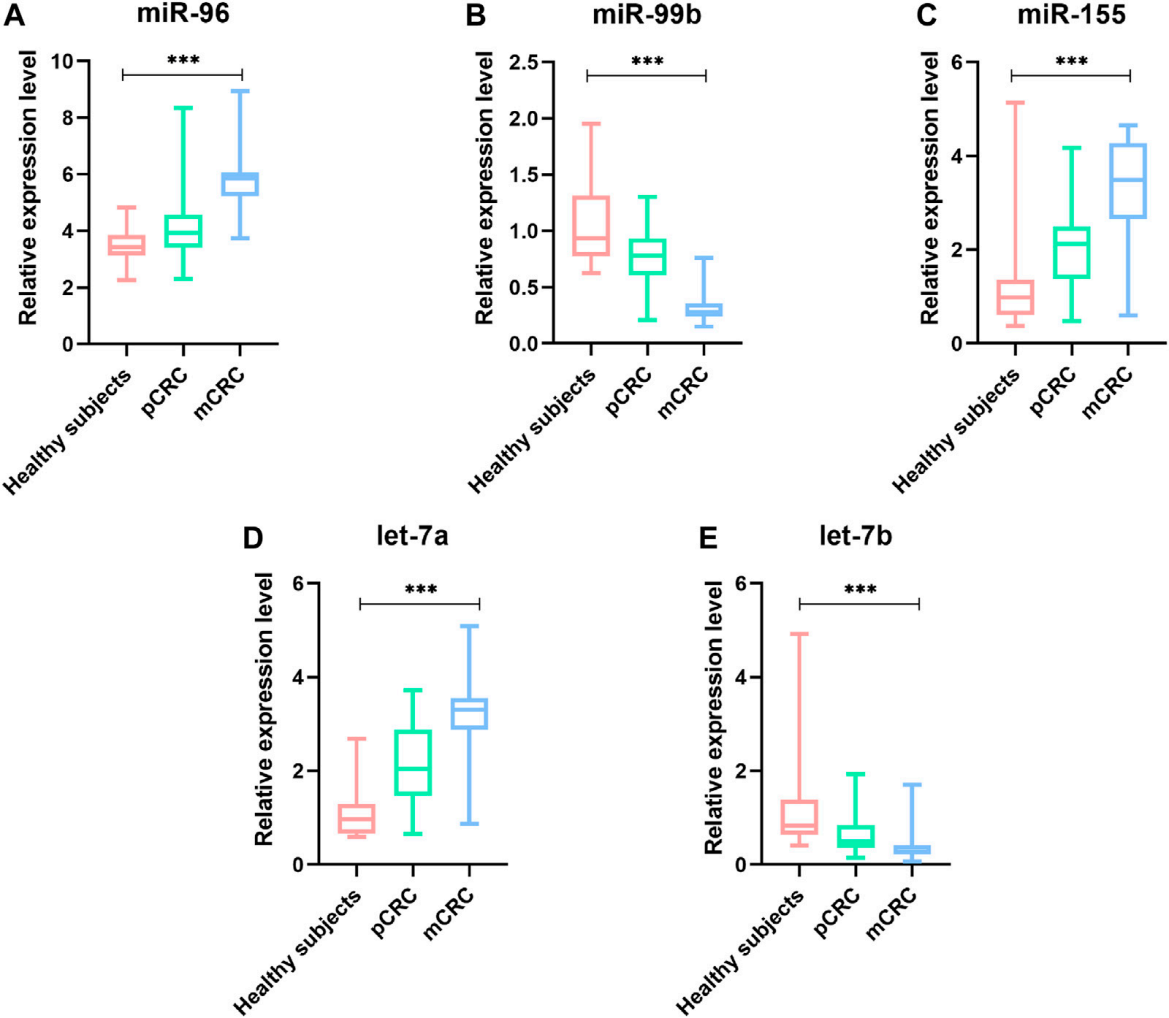
The relative expression level of miRNAs in plasma samples (41 pCRC, 49 mCRC and 20 controls). **(A)** miR-96;**(B)** miR-99b;**(C)** miR-155;**(D)** Let-7a;**(E)** Let-7b; *p value < 0.05, *** means *p* value < 0.001.

To identify whether the plasma levels of miR-96, miR-99b, miR-155, let-7a and let-7b were altered in mCRC patients, qRT-PCR was performed to assess the plasma expression of the five miRNAs in patients and healthy controls. Statistical analysis revealed that miR-96, miR-155 and let-7a were increased, while the miR-99b and let-7b were reduced significantly in CRC patients compared to healthy subjects (Figure 3). Then, we compared the expression levels of plasma miR-96, miR-99b, miR-155, let-7a, and let-7b between pCRC and mCRC patients. These miRNAs are abnormally expressed between CRC patients and controls. Compared with pCRC patients, plasma miR-96, miR-155 and let-7a are up-regulated in mCRC patients, while miR-99b and let-7b are down-regulated. Detailed analysis showed that the expression of plasma miR-96, miR-155 and let-7a gradually increased with the progress of clinical stages, while the expression levels of miR-99b and let-7b showed a downward trend with the progress of clinical stages (Figure 4).

### Survival Analyses for CRC

**FIGURE 5:**
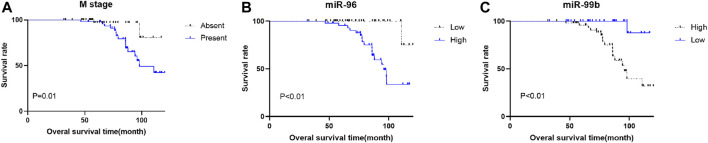
Kaplan–Meier survival curves for CRC patients. Patients were categorized into two groups based on the medium expression levels of miRNAs (*n* = 45 respectively). **(A)** there is a significant difference in OS between patients with and without metastasis. **(B)** different miR-96 expression is significantly correlated with OS. C, different miR-99b expression is significantly correlated with OS.

To evaluate the correlation of the plasma miRNA expression levels and OS of CRC patients, we classified all CRC cases into high and low groups according to the median expression levels of miRNA. Univariate survival analysis demonstrated that M stage, CEA level, miR-96, miR-99b and miR-155 were associated with the OS of CRC patients, Multivariate Cox regression analysis showed that plasma miR-96 (high), low-level miR-99b (low) and M stage (present) are independent poor prognostic indicators for CRC patients (Table 4; Figure 5).

**TABLE 4:** Univariate and multivariate analyses for overall survival of CRC patients with liver metastasis.

Characteristic	Univariate analysis		*p* value	Multivariate analysis		*p* value
	RR	95%CI		RR	95%CI	
Age	0.73	0.39–1.37	0.33	—	—	—
Gender	1.28	0.69–2.37	0.43	—	—	—
Differentiation	1.52	0.73–3.19	0.26	—	—	—
Tumor diameter (cm)	1.24	0.67–2.29	0.49	—	—	—
T stage	8.32	1.14–60.59	0.04	0.67	0.62–7.23	0.74
N stage	4.41	1.36–14.31	0.01	0.14	0.03–0.74	**0.02**
M stage	6.18	2.19–17.37	0.00	0.06	0.01–0.35	**0.00**
Clinical stage	1.8	1.86–98.65	0.08	89.73	3.14–2,565.29	**0.00**
CEA	1.62	0.86–3.06	0.14	—	—	—
miR-96	39.11	5.37–284.59	0.00	69.25	5.31–902.75	**0.00**
miR-99b	0.08	0.03–0.21	0.00	0.16	0.04–0.69	**0.01**
miR-155	2.86	1.43–5.73	0.00	1.19	0.44–3.27	0.73
let-7a	2.44	1.19–5.00	0.015	0.61	0.25–1.46	0.26
let-7b	0.44	0.23–0.87	0.02	1.24	0.46–3.36	0.67

Note: The bold values is that the result of multivariate analysis is significant.

### Establish a Nomogram for Prognostic Evaluation

**FIGURE 6:**
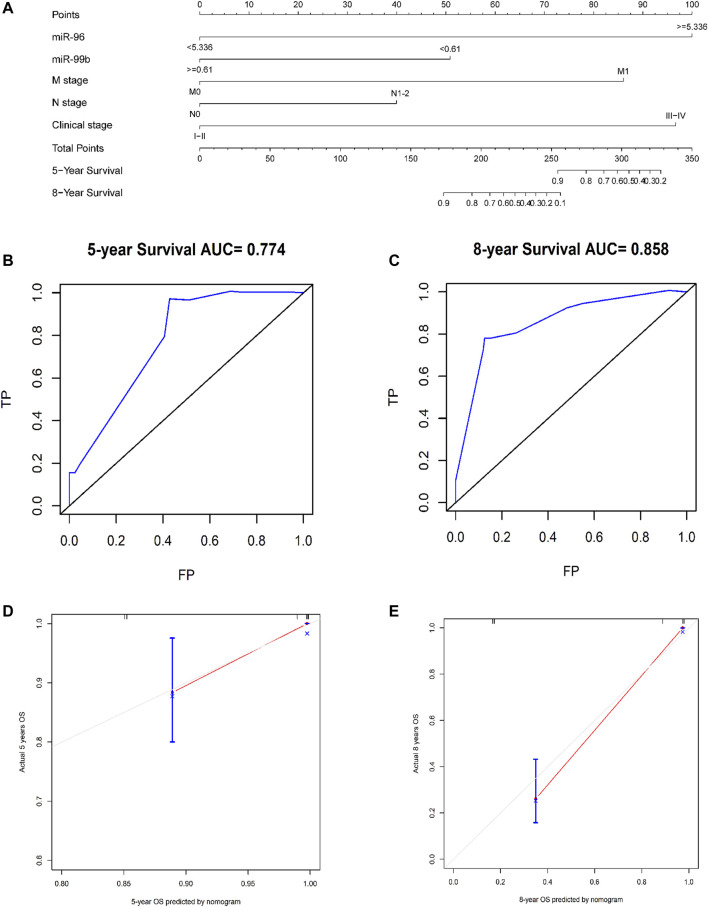
Using nomograms to predict OS in patients with CRC at 5 and 8 years. **(A)** A composite nomogram prognostic model based on miRNAs and clinical parameters. **(B,C)** Use nomogram to perform ROC analysis of OS. **(D,E)** 5-years and 8-years OS calibration curve of nomogram. Note: TP, true possibility; FP, false possibility.

**FIGURE 7:**
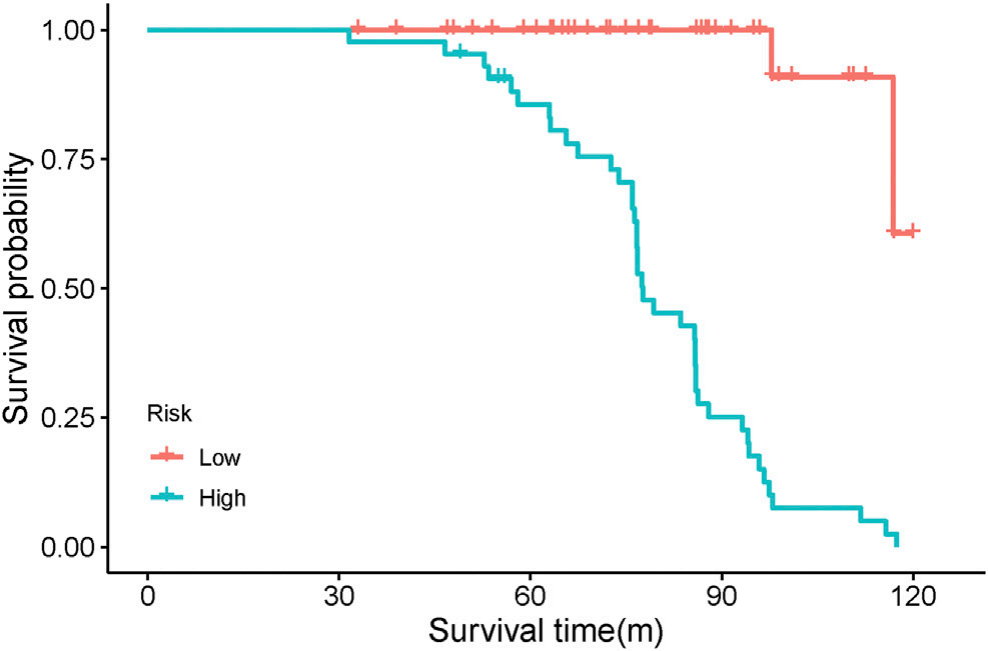
Kaplan-Meier analysis of high-risk and low-risk patients based on risk score.

Based on the five independent prognostic indicators obtained by the multivariate cox regression model, we constructed a nomogram (Figure 6A). The C-index is 0.817, which indicates that the model has a favorable distinguishing ability. The 5-years and 8-years AUC values of the nomogram predicted are 0.77 and 0.86, respectively, indicating that the nomogram is accurate (Figures 6B,C). The calibration curve of the nomogram shows that the predicted survival probability is close to the patients’ actual survival probability, indicating that our model has promising practical application value and prediction accuracy (Figures 6D,E). Then, according to the median risk score, the patients were divided into high- and low-risk groups (Supplementary Figure S1). Kaplan-Meier analysis shows that the survival rate of patients in the high-risk group is lower than that of patients in the low-risk group (*p* < 0.001, Figure 7).

### The Diagnostic Value of miR-96, miR-99b and CEA in Plasma

**FIGURE 8:**
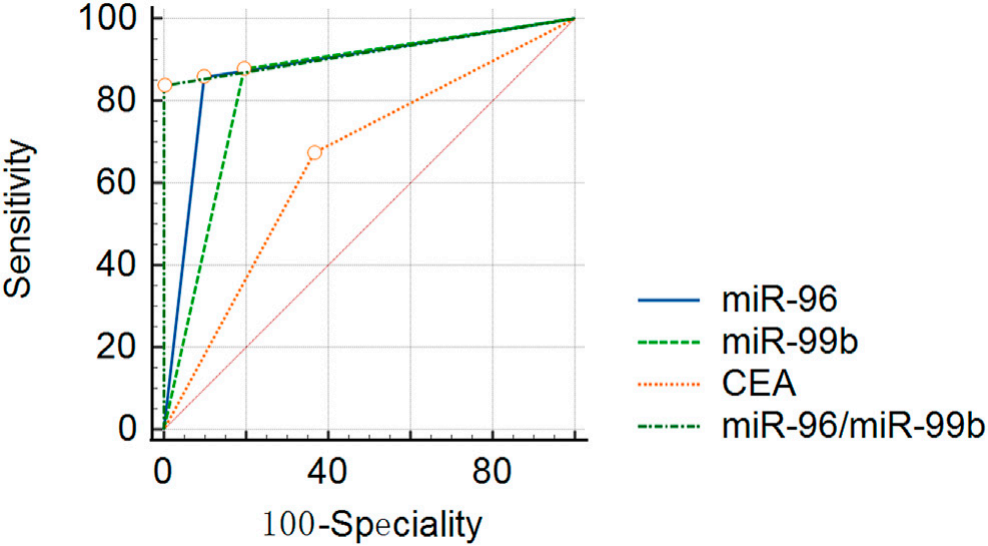
ROC curve analyses in 49 mCRC and 41 pCRC patients. miR-96, miR-99b, CEA and miR-96/miR-99b have satisfactory diagnostic value for mCRC patients. Among them, miR-96/miR-99b is the most powerful combination indicator in our study, with AUC = 0.91.

In order to explore whether the above two prognostic-related miRNAs (high miR-96 and low miR-99b associated with poor OS) have diagnostic value for CRC, we conducted ROC analysis on CRC patients. Considering the actual clinical situation and practicality, we also included CEA and the ratio of miR-96/miR-99b as research indicators. The results demonstrated that the AUC(AUC = 0.93) of miR-96/miR-99b is the highest among all indicators (better than any single indicator and CEA, Table 5; Figure 8). In this way, detecting the expression of miR-96 and miR-99b in plasma can see early CRC patients and have a certain guiding effect on prognosis and treatment. Our results show that the combination of plasma miR-96/miR-99b has excellent discrimination ability and can be adopted as a non-invasive and stable biomarker for the detection and monitoring of mCRC patients.

**TABLE 5:** The diagnostic value of miRNAs between different groups of patients.

pCRC and mCRC	AUC	Cut-off	Sensitivity	Specificity	95%CI	*p* value
miR-96	0.90	0.82	91.84	90.24	0.82–0.95	<0.001
miR-99b	0.91	0.68	95.92	78.05	0.83–0.96	<0.001
miR-96/miR-99b	0.93	0.80	89.80	90.24	0.86–0.97	<0.001
CEA^a^	0.65	0.31	67.35	63.41	0.54–0.75	0.003

Note: ROC, was conducted using MedCalc software.

### GO Annotation and KEGG Enrichment Analyses

**FIGURE 9:**
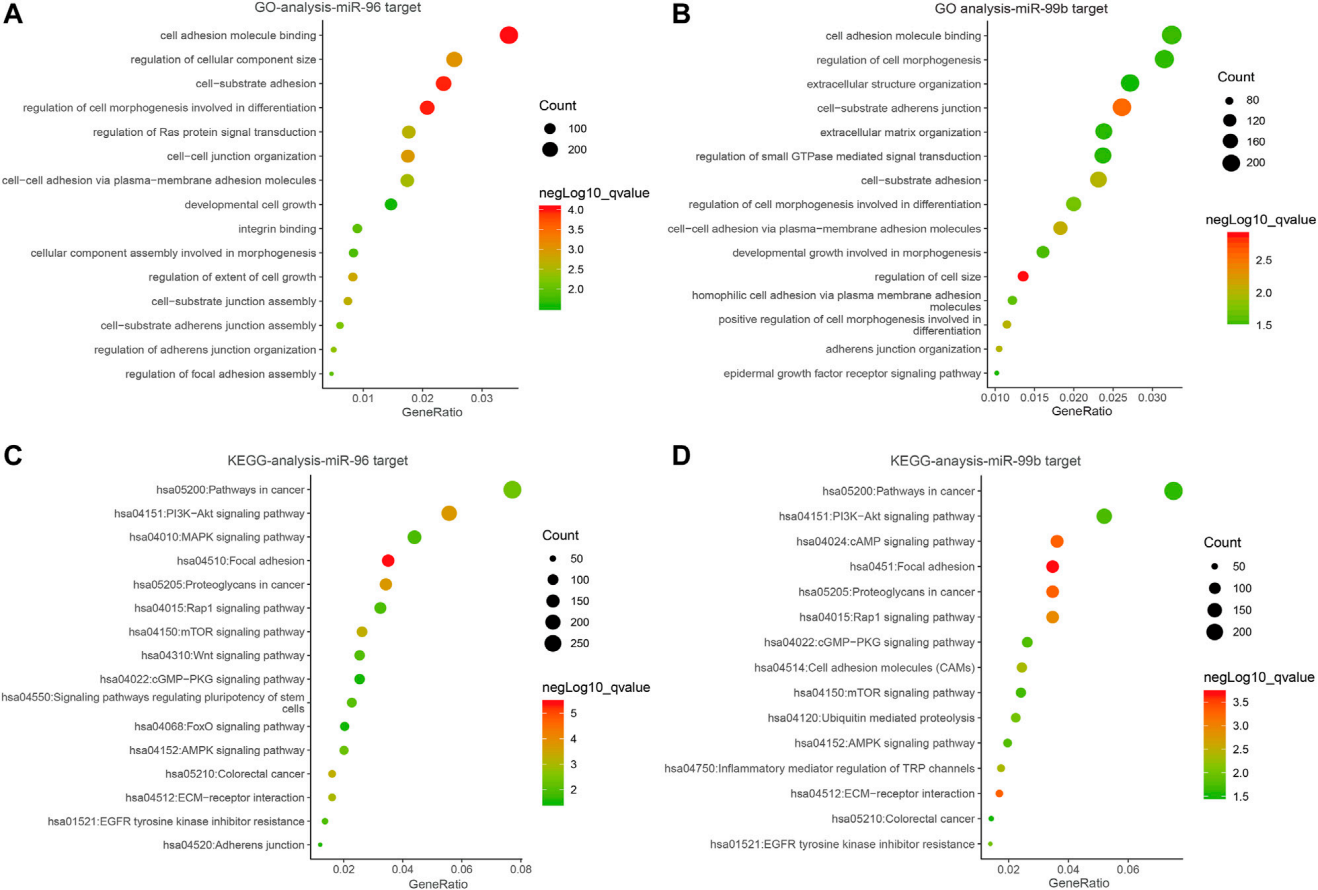
The bubble plot of miRNA bioinformatics analyses. **(A,B)** Gene ontology (GO) analysis; **(C,D)** The KEGG signal pathway analyses.

Several studies have shown the significant function of miRNAs in signaling pathways (Fan et al., 2018). In order to explain the roles of the miR-96 and miR-99b in the development and progress of CRC, we utilized database miRWalk (version.3.0) to identify the most plausible targets of miR-96 and miR-99b. To avoid the missing of crucial information due to merely taking the intersection of the predicted target genes into consideration, a functional enrichment analysis was conducted on all predicted target genes. We eventually obtained 9383 target genes for miR-96 and 8230 genes for miR-99b Supplementary Table S4 and Supplementary Table S5). The target genes of miR-96 are enriched in cancer-related pathways such as PI3K-Akt signaling pathway and mTOR signaling. In miR-99b, the KEGG pathway was enriched in pi3K-Akt and other signaling pathways (Figure 9).

### Construction of PPI Network

**FIGURE 10:**
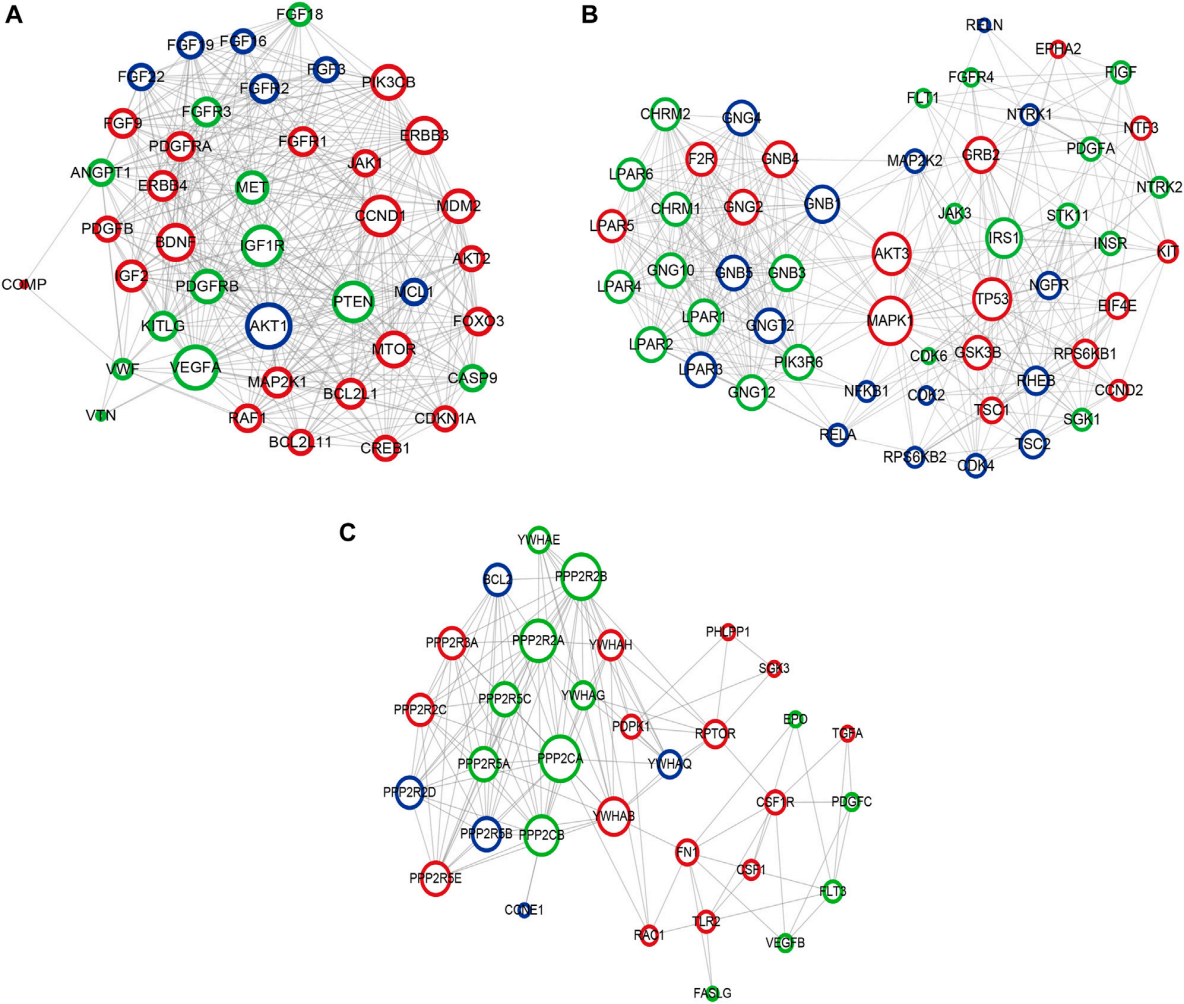
Crucial modules in PPI network of PI3K-Akt signaling pathway. **(A)** module one, **(B)** module two, **(C)** module three. Green circles represent miR-96 target genes, blue circles represent miR-99b target genes, the common target genes are in red.

**FIGURE 11:**
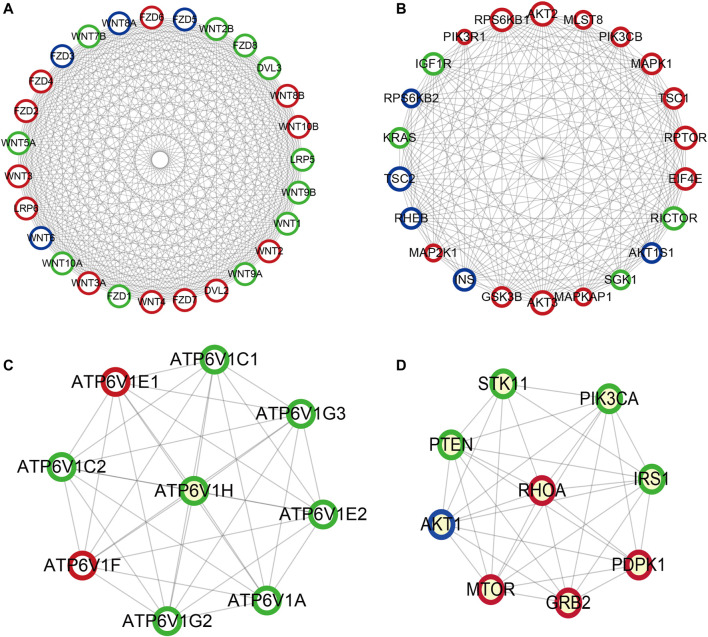
Important modules in PPI network of mTOR signaling pathway **(A)** module one, **(B)** module two, **(C)** module three, **(D)** module four. Green circles represent miR-96 target genes, blue circles represent miR-99b target genes, the common target genes are in red.

Based on the KEGG pathway analyses, PPI network construction uses two common approaches. As shown in Figure 10A, the PPI network of the PI3K-Akt signaling pathway contained 266 nodes and 6308 interaction pairs. Four Module (Figures 10B–E) were obtained, and the details (degrees of each module) are shown in Supplementary Table S6 and Supplementary Table S7. As shown in Figure 11, the PPI network of mTOR signaling pathway contained 121 nodes and 1,647 interaction pairs. We obtained four Module and the degrees of the nodes of each module shown in Supplementary Table S8 and Supplementary Table S9.

## Discussion

The TNM staging system is currently the gold standard for evaluating tumor prognosis, but the impact of genetic changes is not considered. Patients with the same disease stage often have different treatment and prognostic results. Efficient diagnostic methods for the screening of mCRC are the emphasis to better survival. Therefore, identifying new prognostic markers is an ongoing challenge in biomedical research.

Our model is a composite parameter nomogram composed of clinical features and miRNA, rather than just using many clinical parameters or markers (Borumandnia et al., 2021). Moreover, unlike many studies, this study uses the database of the research center to establish a prognostic model and verify it, instead of using online databases such as TCGA (Li et al., 2020). The C-index and calibration chart show that the 5-parameter prediction model has a satisfactory fit and better clinical applicability than the traditional TNM staging system.

Consistent with our findings, accumulating studies reported that miR-96 levels in CRC tissues were higher compared to adjacent normal mucosal tissues (Vega et al., 2013). MiR-96 antagomir may slow down the occurrence of CRC through AMPKα2-related m6A modification, highlighting a new mechanism related to the event of colorectal cancer (Yue et al., 2020). Moreover, Xu et al. suggested that miR-96 was correlated with liver metastasis in CRC patients (Xu et al., 2012). In addition, serum miR-96 also considered an indicator for distinguishing chemoresistance in advanced CRC (Jin et al., 2019).

In contrast with the research mentioned above, some reports have proposed a role of miR-96 as a tumor suppressor (Yue et al., 2020). For instance, Yu et al. suggested that miR-96 functions as a tumor-suppressor miRNA by targeting the KRAS gene in pancreatic cancer (Yu et al., 2010), this may be due to the different pathways where miR-96 works in various cancers. Our data demonstrate that plasma miR-99b was significantly reduced in CRC patients relative to healthy controls. Among the three groups, patients with mCRC (stage IV) showed the lowest expression level of plasma miR-99b, with the healthy individuals exhibiting the highest expression level. Li et al. indicated that miR-99b expression in stage III patients with CRC was significantly higher than patients with mCRC and found miR-99b was more than 6-fold higher in primary colorectal tumor tissues than in matched liver metastases. Slattery et al. identified that miR-99b was associated with survival in patients with colon cancer (Slattery et al., 2016) Zhao YJ et al. suggested that circulating miR-99b can be a diagnostic biomarker for CRC (Zhao et al., 2019), this result is consistent with another study (Jin et al., 2019).

Our research results indicate that miR-96 and miR-99b may participate in the mTOR and PI3K signaling pathways, and then play an important role in the occurrence, development and metastasis of CRC. Many studies have shown that induction of miR-96 can regulate downstream mTOR, thereby promoting tumor metastasis (Siu et al., 2015; Razaviyan et al., 2018). The down-regulation of miR-99b and the up-regulation of its target gene IGF-1R may over-induced the PI3K-AKT signaling pathway, leading to abnormal cell proliferation (Li et al., 2015); miR-99b also attenuates tumor cell migration and invasion by down-regulating the PI3K/AKT/mTOR signaling pathway, providing a therapeutic approach for tumor therapy (Li et al., 2019).

Previous evidence has indicated that plasma miRNAs might derived from the active release of secretory vesicles, necrosis, and apoptotic tumor cells. Therefore, plasma miRNAs could mirror tumor dynamics in different cancer tissues (Kawaguchi et al., 2013; Komatsu et al., 2014). Advanced patients often have a poor prognosis. Therefore, looking for predictive biomarkers that can detect CRC early can provide timely intervention and improve patient survival. If the changes of miR-96/miR-99b can be detected in the blood of patients early, patients with poor prognosis and risk of metastasis can be dynamically monitored and identified as early as possible, and corresponding treatment measures can be taken to reduce the possibility of CRC metastasis.

Our study detected miRNAs from the cell and plasma levels, and proposed a CRC prognosis model based on five indicators: N stage, M stage, clinical stage, and miR-96/miR-99b. However, our study has limitations. This study is a single-center study with small sample size; Our nomogram model still needs internal verification and external verification to improve its accuracy and practicality; In addition, follow-up *in vivo* and *in vitro* studies still needed to verify the results of this study.

In summary, our study proposes a compound-parameter nomogram prognostic model. miR-96 and miR-99b may have prognostic value, but further studies are needed to confirm our research. The PI3K pathway and mTOR pathway frequently dysregulated in CRC patients. Other well-designed, multi-center, large-scale studies should perform to verify our results before clinical application.

## Data Availability

The original contributions presented in the study are included in the article/Supplementary Material, further inquiries can be directed to the corresponding author.

## Abbreviations

miRNAs, MicroRNAs; pCRC, Primary colorectal cancer; mCRC, Metastatic colorectal cancer; OS, Overall survival; ROC, Receiver operating characteristic; qRT-PCR, Quantitative real-time polymerase chain reaction.

## References

[B1] Bartel D. P. (2009). MicroRNAs: Target Recognition and Regulatory Functions. Cell 136, 215–233. 10.1016/j.cell.2009.01.002 19167326 PMC3794896

[B2] Borumandnia N. Doosti H. Jalali A. Khodakarim S. Charati J. Y. Pourhoseingholi M. A. (2021). Nomogram to Predict the Overall Survival of Colorectal Cancer Patients: A Multicenter National Study. Int. J. Environ. Res. Public Health 18. 10.3390/ijerph18157734 PMC8345484 34360026

[B3] Cheng H. Zhang L. Cogdell D. E. Zheng H. Schetter A. J. Nykter M. (2011). Circulating Plasma MiR-141 Is a Novel Biomarker for Metastatic colon Cancer and Predicts Poor Prognosis. PLoS One 6, e17745. 10.1371/journal.pone.0017745 21445232 PMC3060165

[B4] Fan H. Jiang M. Li B. He Y. Huang C. Luo D. (2018). MicroRNA-let-7a Regulates Cell Autophagy by Targeting Rictor in Gastric Cancer Cell Lines MGC-803 and SGC-7901. Oncol. Rep. 39, 1207–1214. 10.3892/or.2018.6194 29328491

[B5] Gu X. Jin R. Mao X. Wang J. Yuan J. Zhao G. (2018). Prognostic Value of miRNA-181a/b in Colorectal Cancer: a Meta-Analysis. Biomarkers Med. 12, 299–308. 10.2217/bmm-2016-0222 28841043

[B6] Hur K. Toiyama Y. Okugawa Y. Ide S. Imaoka H. Boland C. R. (2017). Circulating microRNA-203 Predicts Prognosis and Metastasis in Human Colorectal Cancer. Gut 66, 654–665. 10.1136/gutjnl-2014-308737 26701878 PMC4919275

[B8] Imaoka H. Toiyama Y. Fujikawa H. Hiro J. Saigusa S. Tanaka K. (2016). Circulating microRNA-1290 as a Novel Diagnostic and Prognostic Biomarker in Human Colorectal Cancer. Ann. Oncol. 27, 1879–1886. 10.1093/annonc/mdw279 27502702

[B9] Jin G. Liu Y. Zhang J. Bian Z. Yao S. Fei B. (2019). A Panel of Serum Exosomal microRNAs as Predictive Markers for Chemoresistance in Advanced Colorectal Cancer. Cancer Chemother. Pharmacol. 84, 315–325. 10.1007/s00280-019-03867-6 31089750

[B10] Kanaan Z. Roberts H. Eichenberger M. R. Billeter A. Ocheretner G. Pan J. (2013). A Plasma MicroRNA Panel for Detection of Colorectal Adenomas. Ann. Surg. 258, 400–408. 10.1097/sla.0b013e3182a15bcc 24022433

[B11] Kawaguchi T. Komatsu S. Ichikawa D. Morimura R. Tsujiura M. Konishi H. (2013). Clinical Impact of Circulating miR-221 in Plasma of Patients with Pancreatic Cancer. Br. J. Cancer 108, 361–369. 10.1038/bjc.2012.546 23329235 PMC3566805

[B12] Kent O. A. Mendell J. T. (2006). A Small Piece in the Cancer Puzzle: microRNAs as Tumor Suppressors and Oncogenes. Oncogene 25, 6188–6196. 10.1038/sj.onc.1209913 17028598

[B13] Komatsu S. Ichikawa D. Hirajima S. Kawaguchi T. Miyamae M. Okajima W. (2014). Plasma microRNA Profiles: Identification of miR-25 as a Novel Diagnostic and Monitoring Biomarker in Oesophageal Squamous Cell Carcinoma. Br. J. Cancer 111, 1614–1624. 10.1038/bjc.2014.451 25117812 PMC4200091

[B14] Li J. Fang R. Gong Q. Wang J. (2015). miR-99b Suppresses IGF-1R Expression and Contributes to Inhibition of Cell Proliferation in Human Epidermal Keratinocytes. Biomed. Pharmacother. 75, 159–164. 10.1016/j.biopha.2015.07.013 26297545

[B15] Li W. Yu W. Jiang X. Gao X. Wang G. Jin X. (2020). The Construction and Comprehensive Prognostic Analysis of the LncRNA-Associated Competitive Endogenous RNAs Network in Colorectal Cancer. Front. Genet. 11, 583. 10.3389/fgene.2020.00583 32714366 PMC7344331

[B16] Li Y. J. Wang Y. Wang Y. Y. (2019). Retracted : MicroRNA‐99b Suppresses Human Cervical Cancer Cell Activity by Inhibiting the PI3K/AKT/mTOR Signaling Pathway. J. Cel Physiol 234, 9577–9591. 10.1002/jcp.27645 30480801

[B17] Manfredi S. Lepage C. m. Hatem C. Coatmeur O. Faivre J. Bouvier A.-M. (2006). Epidemiology and Management of Liver Metastases from Colorectal Cancer. Ann. Surg. 244, 254–259. 10.1097/01.sla.0000217629.94941.cf 16858188 PMC1602156

[B18] Metcalfe M. S. Bridgewater F. H. G. Mullin E. J. Maddern G. J. (2004). Useless and Dangerous-fine Needle Aspiration of Hepatic Colorectal Metastases. BMJ 328, 507–508. 10.1136/bmj.328.7438.507 14988193 PMC351851

[B19] Ning T. Li J. He Y. Zhang H. Wang X. Deng T. (2021). Exosomal miR-208b Related with Oxaliplatin Resistance Promotes Treg Expansion in Colorectal Cancer. Mol. Ther. 10.1016/j.ymthe.2021.04.028 PMC8417448 33905821

[B20] O'Connor T. Wilmut I. Taylor J. (2013). Quantitative Evaluation of Reference Genes for Real-Time PCR DuringIn VitroMaturation of Ovine Oocytes. Reprod. Domest. Anim. 48, 477–483. 10.1111/rda.12112 23066791

[B21] Qin Q. Wei F. Zhang J. Li B. (2017). MiR-134 Suppresses the Migration and Invasion of Non-small Cell Lung Cancer by Targeting ITGB1. Oncol. Rep. 37, 823–830. 10.3892/or.2017.5350 28075475

[B22] Radwan E. Shaltout A. S. Mansor S. G. Shafik E. A. Abbas W. A. Shehata M. R. (2021). Evaluation of Circulating microRNAs-211 and 25 as Diagnostic Biomarkers of Colorectal Cancer. Mol. Biol. Rep. 48, 4601–4610. 10.1007/s11033-021-06493-9 34132944

[B23] Raut J. R. Schöttker B. Holleczek B. Guo F. Bhardwaj M. Miah K. (2021). A MicroRNA Panel Compared to Environmental and Polygenic Scores for Colorectal Cancer Risk Prediction. Nat. Commun. 12, 4811. 10.1038/s41467-021-25067-8 34376648 PMC8355103

[B24] Razaviyan J. Hadavi R. Tavakoli R. Kamani F. Paknejad M. Mohammadi-Yeganeh S. (2018). Expression of MiRNAs Targeting mTOR and S6K1 Genes of mTOR Signaling Pathway Including miR-96, miR-557, and miR-3182 in Triple-Negative Breast Cancer. Appl. Biochem. Biotechnol. 186, 1074–1089. 10.1007/s12010-018-2773-8 29862445

[B25] Siu M. K. Tsai Y.-C. Chang Y.-S. Yin J. J. Suau F. Chen W.-Y. (2015). Transforming Growth Factor-β Promotes Prostate Bone Metastasis through Induction of microRNA-96 and Activation of the mTOR Pathway. Oncogene 34, 4767–4776. 10.1038/onc.2014.414 25531317

[B26] Slattery M. L. Herrick J. S. Mullany L. E. Wolff E. Hoffman M. D. Pellatt D. F. (2016). Colorectal Tumor Molecular Phenotype and miRNA: Expression Profiles and Prognosis. Mod. Pathol. 29, 915–927. 10.1038/modpathol.2016.73 27198570 PMC4967007

[B7] Srihari S. Leong H. W. (2012). Temporal Dynamics of Protein Complexes in PPI Networks: A Case Study Using Yeast Cell Cycle Dynamics. BMC Bioinformatics 13 (Suppl 17), S16. 10.1186/1471-2105-13-S17-S16 PMC3521212 23282200

[B27] Sung H. Ferlay J. Siegel R. L. Laversanne M. Soerjomataram I. Jemal A. (2021). Global Cancer Statistics 2020: GLOBOCAN Estimates of Incidence and Mortality Worldwide for 36 Cancers in 185 Countries. CA A. Cancer J. Clin. 71, 209–249. 10.3322/caac.21660 33538338

[B28] Vega A. B. Pericay C. Moya I. Ferrer A. Dotor E. Pisa A. (2013). MicroRNA Expression Profile in Stage III Colorectal Cancer: Circulating miR-18a and miR-29a as Promising Biomarkers. Oncol. Rep. 30, 320–326. 10.3892/or.2013.2475 23673725

[B29] Wang Y. Li J. Xia Y. Gong R. Wang K. Yan Z. (2013). Prognostic Nomogram for Intrahepatic Cholangiocarcinoma after Partial Hepatectomy. Jco 31, 1188–1195. 10.1200/jco.2012.41.5984 23358969

[B30] Xu X. M. Qian J. C. Deng Z. L. Cai Z. Tang T. Wang P. (2012). Expression of miR-21, miR-31, miR-96 and miR-135b Is Correlated with the Clinical Parameters of Colorectal Cancer. Oncol. Lett. 4, 339–345. 10.3892/ol.2012.714 22844381 PMC3402725

[B31] Yang H. Fang F. Chang R. Yang L. (2013). MicroRNA-140-5p Suppresses Tumor Growth and Metastasis by Targeting Transforming Growth Factor β Receptor 1 and Fibroblast Growth Factor 9 in Hepatocellular Carcinoma. Hepatology 58, 205–217. 10.1002/hep.26315 23401231

[B32] Yu S. Lu Z. Liu C. Meng Y. Ma Y. Zhao W. (2010). MiRNA-96 Suppresses KRAS and Functions as a Tumor Suppressor Gene in Pancreatic Cancer. Cancer Res. 70, 6015–6025. 10.1158/0008-5472.can-09-4531 20610624

[B33] Yue C. Chen J. Li Z. Li L. Chen J. Guo Y. (2020). MicroRNA-96 Promotes Occurrence and Progression of Colorectal Cancer via Regulation of the AMPKα2-FTO-m6A/MYC axis. J. Exp. Clin. Cancer Res. 39, 240. 10.1186/s13046-020-01731-7 33183350 PMC7659164

[B34] Zhang Y. Guo L. Li Y. Feng G.-H. Teng F. Li W. (2018). MicroRNA-494 Promotes Cancer Progression and Targets Adenomatous Polyposis Coli in Colorectal Cancer. Mol. Cancer 17, 1. 10.1186/s12943-017-0753-1 29304823 PMC5755155

[B35] Zhao Y. j. Song X. Niu L. Tang Y. Song X. Xie L. (2019). Circulating Exosomal miR-150-5p and miR-99b-5p as Diagnostic Biomarkers for Colorectal Cancer. Front. Oncol. 9, 1129. 10.3389/fonc.2019.01129 31750241 PMC6842995

