# Supplementary figures and images for: The High Ratio of the Plasma miR-96/miR-99b Correlated With Poor Prognosis in Patients With Metastatic Colorectal Cancer
Source: Front Mol Biosci. 2022 Jan 3;8:799060. doi: 10.3389/fmolb.2021.799060 (PMC8762210; doi:10.3389/fmolb.2021.799060)

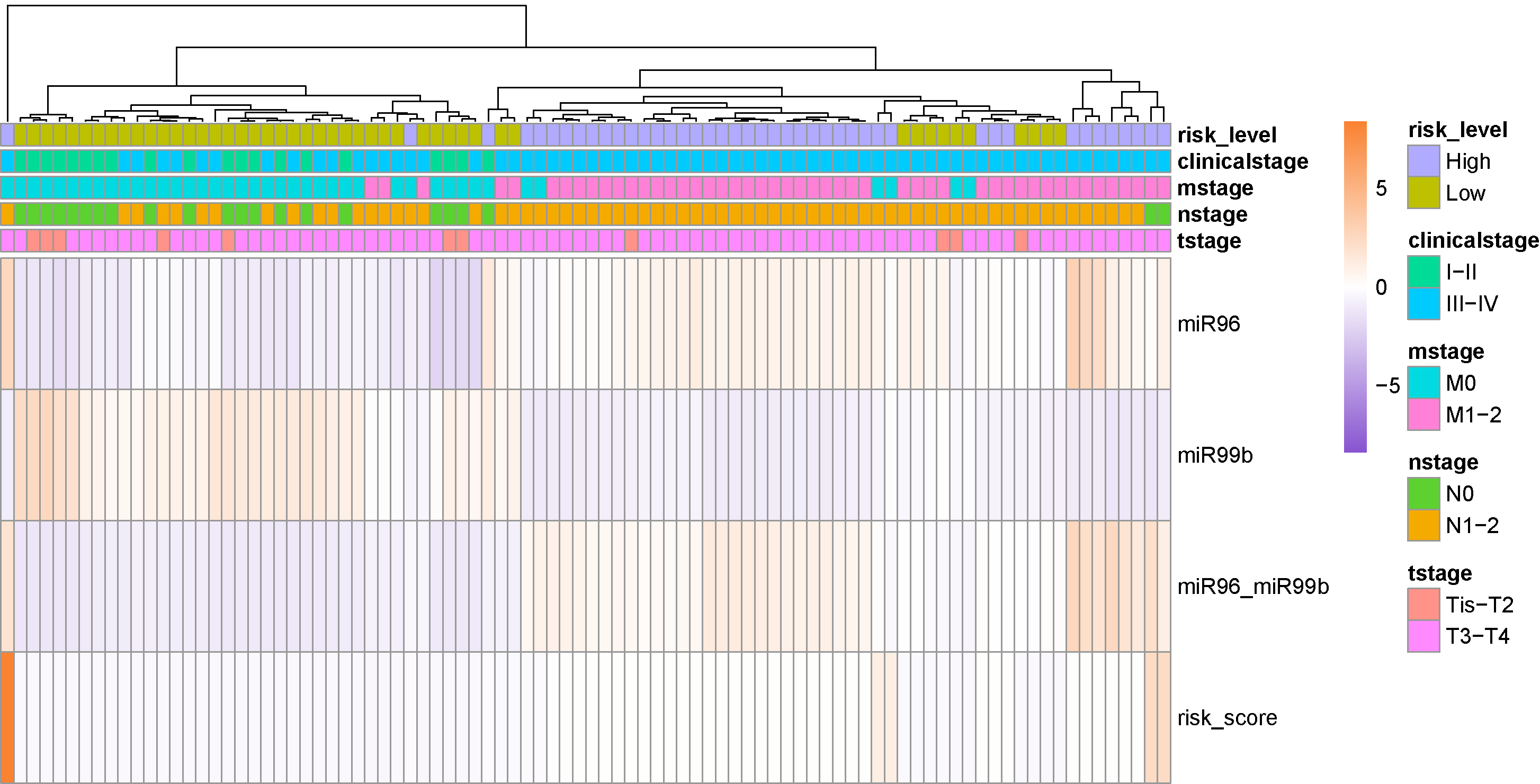

Supplement: Supplementary file 3 [file Image1.TIF]
