# Supplementary material for: The High Ratio of the Plasma miR-96/miR-99b Correlated With Poor Prognosis in Patients With Metastatic Colorectal Cancer
Source: Front Mol Biosci. 2022 Jan 3;8:799060. doi: 10.3389/fmolb.2021.799060 (PMC8762210; doi:10.3389/fmolb.2021.799060)
Supplement: Supplementary file 5 [file Table1.DOC]

**Supplementary table 1. Expression levels of miR-29a,-92a and -16 in plasma samples. Each sample was run in duplicates for analysis.**

| **Cases*** | **miR-29a average(Ct)** | **miR-92a average(Ct)** | **miR-16 average(Ct)** | **2ΔΔct(miR-29a level normalized to miR-16)$** | **2ΔΔct(miR-92a level normalized to miR-16)$** |
| --- | --- | --- | --- | --- | --- |
| CRC015 | 23.34 | 19.36 | 18.52 | 3.864 | 1.223 |
| CRC016 | 26.78 | 18.69 | 19.69 | 0.801 | 4.377 |
| CRC017 | 25.67 | 19.3 | 18.48 | 0.747 | 1.240 |
| CRC018 | 27.55 | 19.68 | 20.21 | 0.674 | 3.160 |
| CRC019 | 28.78 | 20.32 | 20.92 | 0.470 | 3.317 |
| CRC020 | 30.07 | 22.29 | 24.71 | 2.657 | 11.632 |
| CRC021 | 28.01 | 20.21 | 19.6 | 0.321 | 1.424 |
| CRC022 | 29.96 | 22.25 | 24.82 | 3.074 | 12.906 |
| CRC023 | 28.27 | 20.19 | 21.03 | 0.717 | 3.891 |
| CRC024 | 27.05 | 19.83 | 19.45 | 0.563 | 1.670 |
| CRC025 | 24.76 | 18.92 | 22.1 | 17.268 | 19.698 |
| CRC026 | 28.6 | 23.58 | 23.58 | 3.364 | 2.174 |
| CRC027 | 28.05 | 23.27 | 22.55 | 2.412 | 1.320 |
| CRC028 | 26.32 | 19.54 | 19.42 | 0.914 | 2.000 |
| CRC029 | 29.64 | 23.76 | 25.14 | 4.823 | 5.657 |
| CRC030 | 28.28 | 20.52 | 20.44 | 0.476 | 2.071 |
| CRC031 | 27.82 | 20.13 | 21.94 | 1.853 | 7.621 |
| CRC032 | 28.21 | 21.66 | 21.99 | 1.464 | 2.751 |
| CRC033 | 25.73 | 20.51 | 20.99 | 4.084 | 3.053 |
| CRC034 | 28.48 | 22.18 | 24.26 | 5.856 | 9.190 |
| CRC035 | 25.28 | 19.65 | 20.21 | 3.227 | 3.227 |
| CRC036 | 25.53 | 19.32 | 19.4 | 1.558 | 2.313 |
| CRC037 | 27.69 | 21.26 | 21.39 | 1.385 | 2.395 |
| CRC038 | 27.82 | 21.95 | 24.57 | 11.472 | 13.361 |
| CRC039 | 25.11 | 18.83 | 19.65 | 2.479 | 3.837 |
| CRC040 | 27.65 | 19.71 | 22.45 | 2.990 | 14.621 |
| CRC041 | 26.64 | 20.82 | 22.97 | 8.574 | 9.714 |
| CRC042 | 27.9 | 19.66 | 19.44 | 0.310 | 1.866 |
| CRC043 | 27.4 | 19.74 | 18.86 | 0.293 | 1.181 |
| CRC044 | 27.03 | 18.46 | 19.61 | 0.633 | 4.823 |
| CRC045 | 26.57 | 19.68 | 20.08 | 1.214 | 2.888 |
| CRC046 | 29.06 | 20.21 | 21.19 | 0.467 | 4.317 |
| CRC047 | 29.84 | 23.06 | 24.76 | 3.204 | 7.062 |
| CRC048 | 29.6 | 22.44 | 24.89 | 4.141 | 11.876 |
| CRC049 | 25.64 | 19.39 | 19.33 | 1.376 | 2.085 |
| CRC050 | 27.26 | 22.34 | 21.73 | 2.362 | 1.434 |
| CRC051 | 23.59 | 20.92 | 21.32 | 22.627 | 2.868 |
| CRC052 | 25.55 | 20.9 | 20.49 | 3.272 | 1.647 |
| CRC053 | 25.5 | 20.02 | 19.24 | 1.424 | 1.275 |
| CRC054 | 25.82 | 21.22 | 20.53 | 2.790 | 1.357 |
| CRC055 | 26.68 | 22.09 | 22.14 | 4.724 | 2.266 |
| CRC056 | 28.47 | 21.79 | 20.27 | 0.371 | 0.763 |
| CRC057 | 27.35 | 20.89 | 24.32 | 13.361 | 23.425 |
| CRC058 | 26.49 | 20.51 | 19.88 | 1.117 | 1.404 |
| CRC059 | 27.02 | 21.39 | 20.86 | 1.526 | 1.516 |
| CRC060 | 27.98 | 21.55 | 23.07 | 3.630 | 6.277 |
| CRC061 | 27.44 | 21.34 | 20.89 | 1.165 | 1.591 |
| CRC062 | 25.71 | 21.01 | 18.97 | 1.021 | 0.529 |
| CRC063 | 29.01 | 22.83 | 23.47 | 2.362 | 3.411 |
| CRC064 | 29.31 | 23.99 | 23.27 | 1.659 | 1.329 |
| CRC065 | 31.01 | 24.37 | 24.45 | 1.157 | 2.313 |
| CRC066 | 26.81 | 20.21 | 23.09 | 8.282 | 16.111 |
| CRC067 | 28.09 | 24 | 23.76 | 5.426 | 1.840 |
| CRC068 | 26.08 | 22.34 | 20.96 | 3.138 | 0.835 |
| CRC069 | 26.77 | 22.94 | 20.84 | 1.790 | 0.511 |
| CRC070 | 28.43 | 21.32 | 24 | 5.063 | 14.026 |
| CRC071 | 28.24 | 22.83 | 24.62 | 8.815 | 7.516 |
| CRC072 | 28.41 | 24.09 | 23.55 | 3.732 | 1.495 |
| CRC073 | 27.21 | 22.83 | 21.76 | 2.514 | 1.043 |
| CRC074 | 29.34 | 24.07 | 23.12 | 1.454 | 1.765 |
| CRC075 | 29.62 | 23.42 | 20.53 | 0.202 | 0.435 |
| CRC076 | 28.03 | 22.66 | 22.95 | 3.227 | 3.074 |
| CRC077 | 28.18 | 22.57 | 23.48 | 4.199 | 5.816 |
| CRC078 | 28.33 | 23.59 | 24.27 | 6.543 | 5.426 |
| CRC079 | 29.25 | 22.69 | 24.52 | 4.141 | 4.000 |
| CRC080 | 27.63 | 21.8 | 23.64 | 6.869 | 7.210 |
| CRC081 | 26.27 | 19.65 | 24.35 | 28.840 | 77.709 |
| CRC082 | 29.42 | 24.23 | 24.94 | 4.891 | 8.877 |
| CRC083 | 26.57 | 21.6 | 24.31 | 22.785 | 27.284 |
| CRC084 | 28.87 | 25.31 | 23.16 | 2.085 | 1.021 |
| CRC085 | 25.23 | 21.28 | 20.29 | 3.555 | 1.102 |
| CRC086 | 25.81 | 21.45 | 18.44 | 0.655 | 0.272 |
| CRC087 | 24.6 | 20.69 | 19.59 | 3.387 | 1.014 |
| CRC088 | 26.35 | 23.18 | 21.45 | 3.655 | 0.660 |
| CRC089 | 26.96 | 23.26 | 25.31 | 34.776 | 9.001 |
| CRC090 | 28.2 | 24.35 | 25.9 | 22.162 | 6.409 |
| CRC091 | 27.17 | 23.43 | 24.75 | 20.393 | 5.464 |
| CRC092 | 27.3 | 22.27 | 24.92 | 20.966 | 13.642 |
| CRC093 | 24.9 | 19.23 | 19.59 | 2.751 | 2.790 |
| CRC094 | 24.41 | 19.54 | 19.52 | 3.681 | 2.159 |
| CRC095 | 24.63 | 19.4 | 18.65 | 1.729 | 1.301 |
| CRC096 | 22.94 | 18.46 | 18.31 | 4.438 | 1.973 |
| CRC097 | 26.63 | 19.45 | 19.43 | 0.742 | 2.144 |
| CRC098 | 25.72 | 20.77 | 21.03 | 4.228 | 2.621 |
| CRC099 | 27.99 | 22.22 | 24.03 | 7.013 | 7.621 |
| CRC100 | 27.3 | 20.79 | 21.26 | 3.317 | 6.063 |
| CRC101 | 27.94 | 21.16 | 22.18 | 2.014 | 4.408 |
| CRC102 | 28.09 | 21.22 | 22.53 | 2.313 | 5.389 |
| CRC103 | 27.99 | 20.76 | 21.07 | 0.901 | 2.713 |
| CRC104 | 28.89 | 20.77 | 20.11 | 0.248 | 1.376 |
| CRC105 | 26.2 | 19.99 | 21.09 | 3.160 | 4.691 |
| CRC106 | 29.2 | 20.88 | 21.29 | 0.454 | 2.888 |
| CRC107 | 29.98 | 22.04 | 22.65 | 0.674 | 3.317 |
| CRC108 | 28.81 | 20.82 | 24.14 | 4.287 | 21.857 |
| CRC109 | 29.91 | 21.87 | 22.1 | 0.486 | 2.567 |
| CRC110 | 26.6 | 20.46 | 20.05 | 1.165 | 1.647 |
| CRC111 | 28.63 | 21.79 | 20.53 | 0.398 | 0.908 |
| CRC112 | 28.76 | 22.27 | 23.15 | 2.235 | 4.000 |
| CRC113 | 26.88 | 20.28 | 18.83 | 0.412 | 0.796 |
| CRC114 | 26.69 | 20.98 | 19.7 | 0.859 | 0.895 |
| A001 | 29.26 | 22.64 | 23.41 | 1.892 | 3.732 |
| A002 | 28.09 | 19.93 | 21.61 | 1.223 | 6.964 |
| A003 | 27.79 | 22.44 | 21.21 | 1.141 | 0.933 |
| A005 | 28.09 | 22.77 | 20.67 | 0.637 | 0.511 |
| A006 | 27.7 | 21.3 | 20.34 | 0.664 | 1.117 |
| A007 | 27.79 | 20.08 | 21.56 | 1.454 | 6.105 |
| A008 | 27.28 | 20.52 | 20.97 | 1.376 | 2.990 |
| A009 | 27.95 | 23.55 | 21.56 | 1.292 | 0.547 |
| A010 | 26.89 | 21.77 | 19.74 | 0.768 | 0.536 |
| A012 | 27.33 | 20.64 | 18.8 | 0.295 | 0.611 |
| A013 | 24.43 | 19.93 | 19.63 | 3.918 | 1.765 |
| A014 | 26.26 | 20.95 | 21.06 | 2.969 | 2.362 |
| A015 | 27.81 | 19.64 | 19.34 | 0.308 | 1.778 |
| A016 | 28.57 | 20.43 | 19.66 | 0.225 | 1.283 |
| A019 | 27.75 | 21.86 | 19.14 | 0.279 | 0.332 |
| A020 | 29.23 | 21.46 | 19.38 | 0.118 | 0.514 |
| A022 | 26.66 | 20.35 | 19.97 | 1.057 | 1.670 |
| A023 | 24.66 | 20.08 | 19.22 | 2.497 | 1.198 |
| A024 | 26.67 | 20.15 | 20.92 | 2.014 | 3.732 |
| A025 | 26.5 | 20.22 | 20.71 | 1.973 | 3.074 |
| A026 | 24.64 | 18.52 | 19.74 | 3.655 | 5.098 |
| A027 | 25.37 | 19.44 | 19.66 | 2.085 | 2.532 |
| A028 | 27.64 | 22.21 | 25.05 | 18.001 | 15.563 |
| A029 | 27.16 | 22.07 | 24.42 | 16.336 | 11.081 |
| A030 | 24.99 | 21.23 | 20.87 | 6.320 | 1.705 |
| A031 | 26.22 | 21 | 21.44 | 3.972 | 2.949 |
| A032 | 25.89 | 19.55 | 20.01 | 1.853 | 2.990 |
| A033 | 28.65 | 20.92 | 21.32 | 0.683 | 2.888 |
| A034 | 25.75 | 20.01 | 19.95 | 1.959 | 2.099 |
| A035 | 27.25 | 19.6 | 18.48 | 0.250 | 1.007 |
| A036 | 26.89 | 20.03 | 21.24 | 2.174 | 5.063 |
| A037 | 26.82 | 19.32 | 20.66 | 1.526 | 5.502 |
| A038 | 27.97 | 21.65 | 22.43 | 2.362 | 3.758 |
| A039 | 25.67 | 18.95 | 19.22 | 1.240 | 2.639 |
| A040 | 27.21 | 22.87 | 21.39 | 1.919 | 0.779 |
| A041 | 27.78 | 21.69 | 24.28 | 9.647 | 13.178 |
| A042 | 27.96 | 21.98 | 19.75 | 0.369 | 0.463 |
| A043 | 25.94 | 19.85 | 19.16 | 0.986 | 1.347 |
| A044 | 28.03 | 21.23 | 21.18 | 0.946 | 2.099 |
| A045 | 28.27 | 22.11 | 22.5 | 2.000 | 2.868 |
| A046 | 26.77 | 21.99 | 22.63 | 6.190 | 3.387 |
| N011 | 25.11 | 20.52 | 18.85 | 0.707 | 0.683 |
| N012 | 26.54 | 21.18 | 19.87 | 1.072 | 0.883 |
| N013 | 27.53 | 22.12 | 21.73 | 1.959 | 1.659 |
| N014 | 27.56 | 20.25 | 19.39 | 0.379 | 1.206 |
| N015 | 28.02 | 22.31 | 21.48 | 1.173 | 1.223 |
| N016 | 28.67 | 23.12 | 22.47 | 1.485 | 1.395 |
| N017 | 28.02 | 21.18 | 21.54 | 1.223 | 2.809 |
| N018 | 26.82 | 20.99 | 18.88 | 0.441 | 0.504 |
| N019 | 29.94 | 22.01 | 21.06 | 0.232 | 1.125 |
| N020 | 28.18 | 20.94 | 20.69 | 0.607 | 1.840 |
| N021 | 26.42 | 20.17 | 18.8 | 0.551 | 0.841 |
| N022 | 30.87 | 24.46 | 24.46 | 1.283 | 2.189 |
| N023 | 29.47 | 21.82 | 19.76 | 0.130 | 0.521 |
| N024 | 31.02 | 23.9 | 23.64 | 0.655 | 1.828 |
| N025 | 29.76 | 21.97 | 21.4 | 0.332 | 1.474 |
| N026 | 29.88 | 21.83 | 21.09 | 0.245 | 1.310 |
| N027 | 31.46 | 22.55 | 22.63 | 0.240 | 2.313 |
| N028 | 30.64 | 23.43 | 22.21 | 0.316 | 0.940 |
| N029 | 31.15 | 23.99 | 22.48 | 0.268 | 0.763 |
| N030 | 31.14 | 22.73 | 22.42 | 0.259 | 1.753 |
| N031 | 32.83 | 23.49 | 22.15 | 0.067 | 0.859 |
| N032 | 33.56 | 24.55 | 23.36 | 0.093 | 0.959 |
| N033 | 31.6 | 21.59 | 21.19 | 0.080 | 1.647 |
| N034 | 33.87 | 24.32 | 23.8 | 0.101 | 1.516 |
| N035 | 32.6 | 24.29 | 22.29 | 0.086 | 0.547 |
| N036 | 31.39 | 24.27 | 21.58 | 0.122 | 0.337 |
| N037 | 31.53 | 23.54 | 20.82 | 0.065 | 0.330 |
| N038 | 29.69 | 25.62 | 23.18 | 1.198 | 0.403 |
| N039 | 27.88 | 24.17 | 20.89 | 0.859 | 0.224 |
| N040 | 28.27 | 24.15 | 21.35 | 0.901 | 0.314 |
| N041 | 28.25 | 23.93 | 20.77 | 0.611 | 0.243 |
| N042 | 28.54 | 24.6 | 21.12 | 0.642 | 0.196 |
| N043 | 28.39 | 22.81 | 20.41 | 0.432 | 0.412 |
| N044 | 28.59 | 22.48 | 20.9 | 0.529 | 0.727 |
| N045 | 29.35 | 22.74 | 21.38 | 0.435 | 0.853 |
| N046 | 29 | 23.77 | 22.37 | 1.094 | 0.824 |
| N047 | 28.54 | 23.51 | 21.98 | 1.165 | 0.758 |
| N048 | 30.29 | 23.2 | 21.99 | 0.346 | 0.940 |
| N049 | 28.74 | 22.72 | 21 | 0.511 | 0.660 |
| N050 | 30.68 | 25.36 | 23.07 | 0.555 | 0.444 |
| N051 | 26.96 | 21.03 | 19.8 | 0.763 | 0.927 |
| N052 | 30.67 | 24.82 | 23.61 | 0.818 | 0.940 |
| N053 | 30.62 | 26.32 | 25.17 | 2.479 | 0.979 |
| N054 | 29.23 | 24.57 | 23.05 | 1.495 | 0.758 |
| N055 | 29 | 23.5 | 22.32 | 1.064 | 0.966 |
| N056 | 30.28 | 24.71 | 23.27 | 0.847 | 0.801 |
| N057 | 32.28 | 25.54 | 23.36 | 0.225 | 0.480 |
| N058 | 30.3 | 24.27 | 23.13 | 0.758 | 0.986 |
| N059 | 30.56 | 22.57 | 23.63 | 0.895 | 4.595 |
| N060 | 28.33 | 21.3 | 22.97 | 2.676 | 7.062 |
| N061 | 26.53 | 20.32 | 21.46 | 3.227 | 4.500 |
| N062 | 28.8 | 21.9 | 20.7 | 0.398 | 0.853 |
| N063 | 30.96 | 22.68 | 20.76 | 0.092 | 0.540 |
| N064 | 30.8 | 22.13 | 20.13 | 0.067 | 0.497 |
| N065 | 30 | 21.92 | 20.02 | 0.108 | 0.536 |
| N066 | 29.07 | 20.8 | 20.05 | 0.212 | 1.223 |
| N067 | 29.71 | 22.57 | 22.58 | 0.779 | 2.114 |
| N068 | 27.67 | 21.92 | 22.18 | 2.428 | 2.378 |
| N069 | 30.61 | 21.74 | 20.93 | 0.133 | 1.223 |

*CRC: colorectal cancer; A: adevanced adenoma; N: normal control.

$ A pooling plasma from all these cases was used as a calibrator sample

| Supplementary Table 2 Differentially expressed miRNA in human colon cancer compared with its liver metastases in GSE72199 | | |
| --- | --- | --- |
| gene | foldchange |  |
| hsa-miR-551b | 1.312296 | up |
| hsa-miR-936 | 1.274284 | up |
| hsa-miR-3174 | 1.119544 | up |
| hsa-miR-146a | 1.020621 | up |
| hsa-miR-1285 | 0.945843 | up |
| hsa-miR-149* | 0.913007 | up |
| hsa-miR-142-5p | 0.899993 | up |
| hsa-miR-223 | 0.887985 | up |
| hsa-miR-513b | 0.837228 | up |
| hsa-miR-lOb | 0.754512 | up |
| jcv-miR-Jl-5p | 0.750322 | up |
| hsa-miR-494 | 0.731083 | up |
| hsa-miR-125a-3p | 0.730299 | up |
| hsa-miR-199a-3p | 0.730114 | up |
| hsa-let-7i | 0.685427 | up |
| hsa-miR-497 | 0.625685 | up |
| hsa-miR-381 | 0.609518 | up |
| hsa-miR-1274a | 0.597932 | up |
| hsa-miR-337-5p | 0.568264 | up |
| hsa-miR-154 | 0.554891 | up |
| hsa-miR-1274b | 0.543264 | up |
| hsa-miR-132 | 0.535788 | up |
| hsa-miR-3195 | 0.530642 | up |
| hsa-miR-127-3p | 0.48599 | up |
| hsa-miR-409-3p | 0.480208 | up |
| hsa-miR-22 | 0.465164 | up |
| hsa-miR-4286 | 0.440413 | up |
| hsa-miR-212 | 0.436136 | up |
| hsa-miR-550a | 0.396222 | up |
| hsa-miR-939 | 0.391443 | up |
| hsa-miR-3614-5p | 0.300615 | up |
| hsa-miR-33b* | 0.289984 | up |
| hsa-miR-664 | -0.31863 | down |
| hsa-miR-5 05 | -0.38737 | down |
| hsa-miR-128 | -0.40766 | down |
| hsa-miR-320c | -0.43551 | down |
| hsa-miR-148 b | -0.51217 | down |
| hsa-miR-98 | -0.51274 | down |
| hsa-miR-200c | -0.52989 | down |
| hsa-miR-7-1 * | -0.58893 | down |
| hsvl-miR-Hl* | -0.62097 | down |
| hsa-miR-200a | -0.63855 | down |
| hsa-miR-29a* | -0.69557 | down |
| hsa-miR-96 | -0.70432 | down |
| hsa-miR-429 | -0.74911 | down |
| hsa-miR-192* | -0.76874 | down |
| hsa-miR-106b* | -0.8446 | down |
| hsa-miR-210 | -0.84698 | down |
| hsa-miR-598 | -0.87103 | down |
| hsa-miR-191 | -0.89405 | down |
| hsa-miR-181d | -0.89785 | down |
| hsa-miR-7 | -0.92823 | down |
| hsa-miR-20a* | -0.93143 | down |
| hsa-miR-3189 | -0.94779 | down |
| hsa-miR-183 | -0.95729 | down |
| hsvl -miR-H8 | -0.96162 | down |
| hsa-miR-203 | -0.97528 | down |
| hsa-miR-432 | -1.01261 | down |
| hsa-miR-195* | -1.03305 | down |
| ebv-miR-BART4 | -1.0841 | down |
| hsa-miR-1180 | -1.12151 | down |
| hsa-miR-1290 | -1.17811 | down |
| hsa-miR-449b* | -1.19808 | down |
| hsa-miR-375 | -1.20615 | down |
| hsa-miR-181c* | -1.22109 | down |
| ebv-miR-BART12 | -1.24266 | down |
| hsa-miR-3148 | -1.25342 | down |
| hsa-miR-1228* | -1.30343 | down |
| hsa-miR-19b-l* | -1.33026 | down |
| hsa-miR-1247 | -1.33521 | down |
| hsa-miR-595 | -1.37846 | down |
| hsa-miR-744* | -1.38283 | down |
| ebv-miR-BARTIO | -1.38421 | down |
| hsa-miR-885-3p | -1.4416 | down |
| hsa-miR-3130-5p | -1.46468 | down |
| hsa-miR-3692* | -1.46658 | down |
| hsa-miR-1258 | -1.54516 | down |
| hsa-miR-514b-3p | -1.83916 | down |
| hsa-miR-205* | -1.86067 | down |
| hsa-miR-885-5p | -2.00695 | down |
| hsa-miR-122* | -2.82073 | down |
| hsa-miR-122 | -4.33787 | down |
| hsa-miR-127-3p | 27.01 | up |
| hsa-miR-135a-3p | 25.25 | up |
| hsa-miR-151a-3p | 2.05 | up |
| hsa-miR-155-5p | 2.64 | up |
| hsa-miR-339-3p | 6.25 | up |
| hsa-miR-3 76a-3p | 33.43 | up |
| hsa-miR-409-3p | 13.71 | up |
| hsa-miR-410 | 10.15 | up |
| hsa-miR-4291 | 2.13 | up |
| hsa-miR-432-5p | 11.32 | up |
| hsa-miR-4478 | 8.98 | up |
| hsa-miR-4673 | 10.56 | up |
| hsa-miR-500a-3p | 80.42 | up |
| hsa-miR-503-5p | 3.24 | up |
| hsa-miR-532-3p | 10.29 | up |
| hsa-miR-542-5p | 2.48 | up |
| hsa-miR-55 lb-3p | 53.53 | up |
| hsa-miR-652-3p | 2.48 | up |
| hsa-miR-654-3p | 10.57 | up |
| hsa-miR-6717-5p | 2.19 | up |
| hsa-miR-96-5p | 11.12 | up |
| hsa-miR-122-3p | 4.97 | down |
| hsa-miR-193b-5p | 2.15 | down |
| hsa-miR-3 78a-3p | 3.67 | down |
| hsa-miR-378d | 123.55 | down |
| hsa-miR-378i | 3.79 | down |
| hsa-miR-4270 | 3.01 | down |
| hsa-miR-4286 | 6.45 | down |
| hsa-miR-4508 | 2.13 | down |
| hsa-miR-4649-3p | 2.15 | down |
| hsa-miR-4701 -5p | 2.31 | down |
| hsa-miR-5585-3p | 2.13 | down |
| hsa-miR-99a-5p | 4.45 | down |

| Supplementary Table 3 The expression of miR-96、miR-99b、miR-155、let-7a and let-7b in literature. | | | | | |  |
| --- | --- | --- | --- | --- | --- | --- |
| miRNA | Tumor | Methods | Expression | Function | References |  |
| miR-96 | Colorectal cancer | Genetic testing.RT-PCR | up | Associated with the MMR | PMID: 19922656 |  |
|  | Colorectal cancer | miRNA chip,RT-PCR | up | related to tumor stage, prognosis and treatment | PMID: 26863633 |  |
|  | Lung cancer | Cell viability assay, cell migration assay,RT-PCR,WB, transfection, luciferase assay | up | Tumor proliferation and migration can be promoted by regulating LM07. | PMID: 28026121 |  |
|  | Prostate cancer | RT-PCR, proliferation assay,WB | up | It was associated with stage, lymph node metastasis and drug resistance | PMID: 23951320 |  |
|  | Breast cancer | miRNA chip,RT-PCR | up | a biomarker for early diagnosis of breast cancer | PMID: 26056355 |  |
|  | Colorectal cancer | RT-PCR | up | Involves in the pathophysiological process of Colorectal cancer | PMID: 25925209 |  |
|  | Colorectal cancer | RT-PCR,ROC | up | It was related to clinical stage, tumor recurrence and prognosis | PMID: 27044381 |  |
|  | Colorectal cancer | Cell proliferation assay,RT-PCR clone formation assay, cell cycle assay,WB assay, luciferase assay | up | By regulating the expression of P53, F0x01 and F0x03A genes, proliferation and metastasis were promoted | PMID: 25369914 |  |
|  | Prostate cancer | miRNA chip,RT-PCR | up | It is related to tumor recurrence after operation and has diagnostic and prognostic value | PMID: 19676045 |  |
|  | Colorectal cancer | RT-PCR | up | Involved in tumor development | PMID: 22844381 |  |
|  | Colorectal cancer | miRNA chip,RT-PCR | up | / | PMID: 23673725 |  |
|  | Colorectal cancer | Gene mutation detection,RT-PCR,WB, transfection, proliferation cycle determination, clone formation assay, apoptosis assay | down | Regulates cell growth and is associated with prognosis | PMID: 25256312 |  |
|  | Pancreatic cancer | RT-PCR, transfection, proliferation, apoptosis and cycle assay, cell colony formation, cell migration and invasion assay, immunohistochemistry, in vitro assay | down | Through the regulation of KRAS gene expression, the metastasis was inhibited | PMID: 20610624 |  |
| miR-99a/b | Breast cancer | RT-PCR, luciferase assay, transfection,WB, cell cycle assay, cell scratch and adhesion assay | / | The interstitial transformation of breast epithelial cells induced by regulating TGF- B promotes the proliferation and migration of breast cancer cells | PMID: 22299047 |  |
|  | Esophagus cancer | RT-PCR | up | molecular marker of esophageal cancer | PMID: 23761828 |  |
|  | Hepatic carcinoma | RT-PCR, transfection, luciferase assay, invasion assay,WB, immunohistochemistry, migration assay | up | By regulating the high expression of CLDNU, tumor metastasis can be promoted, which is related to the difference of 0S and DFS | PMID: 26134929 |  |
|  | Prostate cancer | MRNA microarray, deep sequencing, transfection,WB,PCR, clone formation, deep sequencing, luciferase reporting assay, ELISA | down | Inhibits the occurrence and progression of tumors | PMID: 21212412 |  |
|  | Lung cancer | Transfection,RT-PCR,miRNA microarray, luciferase assay,WB, clone formation, cell proliferation assay | down | By modulating FGFR3, tumor progression was inhibited | PMID: 22969861 |  |
| miR-155 | Colorectal cancer | Transfection, cell proliferation assay. | up | Promote the proliferation, invasion and metastasis of Colorectal cancer cells | PMID: 26261588 |  |
|  | Lung cancer | RT-PCR, cell invasion assay | up | By regulating S0CSLS0CS6 and PTEN, it promotes the development of NSCLC | PMID: 27811366 |  |
|  | Lung cancer | RT-PCR, transfection,WB, cell proliferation assay, cell cycle assay, luciferase assay | up | Promote the proliferation of NSCLC cells by inhibiting FOXOL | PMID: 26548866 |  |
|  | Colorectal cancer | RT-PCR | up | It can be used as a marker for diagnosis and prognosis of Colorectal cancer | PMID: 25528214 |  |
|  | Colorectal cancer | RT-PCR | up | Involves in the pathophysiological process of Colorectal cancer | PMID: 25925209 |  |
|  | Gastric cancer | Transfection, cell proliferation assay. RT-PCR, invasion assay, apoptosis assay, cell cycle assay,WB | down | It plays an antitumor role by regulating cyclin D1 | PMID: 26955820 |  |
| let-7a | Colorectal cancer | RT-PCR, genetic testing, survival analysis | / | And the efficacy of anti-EGFR therapy | PMID: 22584434 |  |
|  | Colorectal cancer | RT-PCR, immunohistochemistry, prognostic characteristic spectrum analysis | up | Through the regulation of KRAS gene expression, promote metastasis | PMID: 22120473 |  |
|  | Colorectal cancer | Cell viability detection, semi-quantitative RT-PCR transfection,WB | down | Promote the growth of Colorectal cancer cells | PMID: 16651716 |  |
|  | Colorectal cancer | Cell transfection,RT-PCR,CCK-8, apoptosis assay, in vitro matrix gel invasion assay,WB, luciferase assay | down | The growth and metastasis of tumor cells were inhibited by the regulation of RTKN | PMID: 27498032 |  |
|  | Colorectal cancer | RT-PCR, tissue microarray, immunohistochemistry, gene mutation detection, laser capture microdissection | down | Inhibition of anti-tumor immune response in patients with Colorectal cancer | PMID: 27737877 |  |
|  | gastric cancer | RT-PCR, cell transfection,WB, cell proliferation, clone formation assay, cell migration and scratch assay, cell invasion assay, in vitro assay | down | Cell growth, migration and invasion were inhibited by PKM2 | PMID: 26745603 |  |
|  | Colorectal cancer | WB, invasion and migration assay, soft AGAR assay,RNA immunoprecipitation and RT-qPCR, luciferase assay | down | By regulating the expression of KRAS and LIN28 genes, metastasis was inhibited | PMID: 21625210 |  |
|  | Colorectal cancer | miRNA chip,RT-qPCR | down | It can be used as a noninvasive biomarker for early diagnosis of Colorectal cancer | PMID: 26793011 |  |
| let'7b | Colorectal cancer | Cell viability detection, semi-quantitative RT-PCR, transfection,WB | down | Promote the growth of Colorectal cancer cells | PMID: 16651716 |  |
|  | Glioma | Transfection, cell proliferation, clone formation, invasion assay, RT-PCR, WB. In vitro assay, immunohistochemistry | down | Through the regulation of the expression of LIN28 gene, and then inhibit the metastasis | PMID: 23846349 |  |
|  | Colorectal cancer | WB, invasion and migration assay, soft AGAR assay,RNA immunoprecipitation and RT-qPCR, luciferase assay | down | By regulating the expression of KRAS and LIN28 genes, metastasis was inhibited | PMID: 21625210 |  |
|  | Melanoma | RT-qPCR, luciferase assay, cell cycle and clone formation | down | Growth and proliferation are inhibited by the regulation of cyclins Di, D3 and A | PMID: 18379589 |  |
|  |  |  |  |  |  |  |

| Supplementary table 6_PI3K pathway——PPI | | | | | | | | | | |  |  |  |  |  |  |  |
| --- | --- | --- | --- | --- | --- | --- | --- | --- | --- | --- | --- | --- | --- | --- | --- | --- | --- |
| Genesymbol | degree |  | module1 | Genesymbol | degree |  | module2 | Genesymbol | degree |  | module3 | Genesymbol | degree |  | module4 | Genesymbol | degree |
| AKT1 | 183 |  |  | COL1A1 | 44 |  |  | VWF | 13 |  |  | AKT3 | 26 |  |  | BCL2 | 11 |
| EGFR | 144 |  |  | PIK3R1 | 26 |  |  |  |  |  |  |  |  |  |  |  |  |
| PIK3CA | 139 |  |  |  |  |  |  |  |  |  |  |  |  |  |  |  |  |
| INS | 136 |  |  |  |  |  |  |  |  |  |  |  |  |  |  |  |  |
| EGF | 135 |  |  | PIK3CA | 26 |  |  |  |  |  |  |  |  |  |  |  |  |
| KRAS | 132 |  |  |  |  |  |  |  |  |  |  |  |  |  |  |  |  |
| MAPK1 | 128 |  |  |  |  |  |  |  |  |  |  |  |  |  |  |  |  |
| PIK3R1 | 126 |  |  |  |  |  |  |  |  |  |  |  |  |  |  |  |  |
| FN1 | 125 |  |  |  |  |  |  |  |  |  |  |  |  |  |  |  |  |
| VEGFA | 118 |  |  |  |  |  |  |  |  |  |  |  |  |  |  |  |  |
| TP53 | 116 |  |  |  |  |  |  |  |  |  |  |  |  |  |  |  |  |
| MTOR | 104 |  |  |  |  |  |  |  |  |  |  |  |  |  |  |  |  |
| PTK2 | 103 |  |  |  |  |  |  |  |  |  |  |  |  |  |  |  |  |
| PTEN | 102 |  |  |  |  |  |  |  |  |  |  |  |  |  |  |  |  |
| ERBB2 | 96 |  |  |  |  |  |  |  |  |  |  |  |  |  |  |  |  |
| AKT3 | 95 |  |  | COL2A1 | 37 |  |  | VEGFA | 33 |  |  | CDK2 | 7 |  |  | CSF1 | 5 |
| AKT2 | 94 |  |  | COL1A2 | 43 |  |  | VTN | 4 |  |  | CCND2 | 9 |  |  | CCNE1 | 2 |
| ITGB1 | 94 |  |  |  |  |  |  |  |  |  |  |  |  |  |  |  |  |
| FGF2 | 93 |  |  |  |  |  |  |  |  |  |  |  |  |  |  |  |  |
| IRS1 | 93 |  |  |  |  |  |  |  |  |  |  |  |  |  |  |  |  |
| ITGB3 | 90 |  |  |  |  |  |  |  |  |  |  |  |  |  |  |  |  |
| HGF | 88 |  |  |  |  |  |  |  |  |  |  |  |  |  |  |  |  |
| IGF1R | 88 |  |  |  |  |  |  |  |  |  |  |  |  |  |  |  |  |
| GRB2 | 87 |  |  |  |  |  |  |  |  |  |  |  |  |  |  |  |  |
| SOS1 | 87 |  |  |  |  |  |  |  |  |  |  |  |  |  |  |  |  |
| CCND1 | 86 |  |  | COL9A1 | 34 |  |  | MAP2K1 | 21 |  |  | GNB1 | 22 |  |  | PPP2R2A | 16 |
| PIK3CB | 86 |  |  |  |  |  |  |  |  |  |  |  |  |  |  |  |  |
| PDGFRB | 84 |  |  |  |  |  |  |  |  |  |  |  |  |  |  |  |  |
| ITGA2B | 81 |  |  |  |  |  |  |  |  |  |  |  |  |  |  |  |  |
| ITGAV | 79 |  |  |  |  |  |  |  |  |  |  |  |  |  |  |  |  |
| NGF | 79 |  |  |  |  |  |  |  |  |  |  |  |  |  |  |  |  |
| ITGA6 | 78 |  |  |  |  |  |  |  |  |  |  |  |  |  |  |  |  |
| KIT | 78 |  |  |  |  |  |  |  |  |  |  |  |  |  |  |  |  |
| PDGFB | 78 |  |  |  |  |  |  |  |  |  |  |  |  |  |  |  |  |
| MAP2K1 | 77 |  |  |  |  |  |  |  |  |  |  |  |  |  |  |  |  |
| MET | 77 |  |  |  |  |  |  |  |  |  |  |  |  |  |  |  |  |
| KDR | 76 |  |  |  |  |  |  |  |  |  |  |  |  |  |  |  |  |
| GSK3B | 75 |  |  |  |  |  |  |  |  |  |  |  |  |  |  |  |  |
| ITGA2 | 75 |  |  |  |  |  |  |  |  |  |  |  |  |  |  |  |  |
| FOXO3 | 74 |  |  |  |  |  |  |  |  |  |  |  |  |  |  |  |  |
| INSR | 74 |  |  |  |  |  |  |  |  |  |  |  |  |  |  |  |  |
| RAF1 | 72 |  |  |  |  |  |  |  |  |  |  |  |  |  |  |  |  |
| RPS6KB1 | 72 |  |  |  |  |  |  |  |  |  |  |  |  |  |  |  |  |
| ITGB4 | 71 |  |  |  |  |  |  |  |  |  |  |  |  |  |  |  |  |
| PDGFRA | 71 |  |  |  |  |  |  |  |  |  |  |  |  |  |  |  |  |
| BDNF | 70 |  |  | COL6A5 | 35 |  |  | MDM2 | 24 |  |  | FIGF | 10 |  |  | PPP2CA | 18 |
| ERBB3 | 70 |  |  |  |  |  |  |  |  |  |  |  |  |  |  |  |  |
| ITGA1 | 70 |  |  |  |  |  |  |  |  |  |  |  |  |  |  |  |  |
| ITGA5 | 69 |  |  |  |  |  |  |  |  |  |  |  |  |  |  |  |  |
| PIK3R3 | 68 |  |  |  |  |  |  |  |  |  |  |  |  |  |  |  |  |
| JAK1 | 67 |  |  |  |  |  |  |  |  |  |  |  |  |  |  |  |  |
| PRKCA | 67 |  |  |  |  |  |  |  |  |  |  |  |  |  |  |  |  |
| IGF2 | 66 |  |  |  |  |  |  |  |  |  |  |  |  |  |  |  |  |
| ITGA4 | 65 |  |  |  |  |  |  |  |  |  |  |  |  |  |  |  |  |
| RAC1 | 65 |  |  |  |  |  |  |  |  |  |  |  |  |  |  |  |  |
| BCL2L1 | 64 |  |  | COL6A2 | 40 |  |  | MTOR | 26 |  |  | F2R | 19 |  |  | PDPK1 | 7 |
| MDM2 | 64 |  |  |  |  |  |  |  |  |  |  |  |  |  |  |  |  |
| NGFR | 64 |  |  |  |  |  |  |  |  |  |  |  |  |  |  |  |  |
| PIK3CD | 64 |  |  |  |  |  |  |  |  |  |  |  |  |  |  |  |  |
| ITGA3 | 63 |  |  |  |  |  |  |  |  |  |  |  |  |  |  |  |  |
| PDPK1 | 63 |  |  |  |  |  |  |  |  |  |  |  |  |  |  |  |  |
| CREB1 | 62 |  |  | ITGB8 | 46 |  |  |  |  |  |  | PIK3R6 | 20 |  |  |  |  |
| ITGA9 | 62 |  |  |  |  |  |  |  |  |  |  |  |  |  |  |  |  |
| ERBB4 | 60 |  |  |  |  |  |  |  |  |  |  |  |  |  |  |  |  |
| FGF1 | 60 |  |  |  |  |  |  |  |  |  |  |  |  |  |  |  |  |
| ITGB5 | 60 |  |  |  |  |  |  |  |  |  |  |  |  |  |  |  |  |
| LAMB1 | 60 |  |  |  |  |  |  |  |  |  |  |  |  |  |  |  |  |
| VWF | 60 |  |  |  |  |  |  |  |  |  |  |  |  |  |  |  |  |
| YWHAB | 60 |  |  |  |  |  |  |  |  |  |  |  |  |  |  |  |  |
| COL1A1 | 59 |  |  | ITGA11 | 46 |  |  | FGF18 | 15 |  |  | JAK3 | 8 |  |  | TLR2 | 6 |
| ITGB6 | 59 |  |  |  |  |  |  |  |  |  |  |  |  |  |  |  |  |
| TSC2 | 59 |  |  |  |  |  |  |  |  |  |  |  |  |  |  |  |  |
| FGFR1 | 58 |  |  |  |  |  |  |  |  |  |  |  |  |  |  |  |  |
| FGFR2 | 58 |  |  |  |  |  |  |  |  |  |  |  |  |  |  |  |  |
| FGFR3 | 58 |  |  |  |  |  |  |  |  |  |  |  |  |  |  |  |  |
| ITGA11 | 58 |  |  |  |  |  |  |  |  |  |  |  |  |  |  |  |  |
| KITLG | 58 |  |  |  |  |  |  |  |  |  |  |  |  |  |  |  |  |
| ITGA7 | 57 |  |  |  |  |  |  |  |  |  |  |  |  |  |  |  |  |
| ITGA8 | 57 |  |  |  |  |  |  |  |  |  |  |  |  |  |  |  |  |
| ITGB8 | 57 |  |  |  |  |  |  |  |  |  |  |  |  |  |  |  |  |
| THBS1 | 57 |  |  |  |  |  |  |  |  |  |  |  |  |  |  |  |  |
| ANGPT1 | 56 |  |  | COL4A1 | 46 |  |  | RAF1 | 19 |  |  | CDK4 | 11 |  |  | CSF1R | 7 |
| EPO | 56 |  |  |  |  |  |  |  |  |  |  |  |  |  |  |  |  |
| ITGA10 | 56 |  |  |  |  |  |  |  |  |  |  |  |  |  |  |  |  |
| PPP2CA | 56 |  |  |  |  |  |  |  |  |  |  |  |  |  |  |  |  |
| RELA | 56 |  |  |  |  |  |  |  |  |  |  |  |  |  |  |  |  |
| FLT1 | 55 |  |  |  |  |  |  |  |  |  |  |  |  |  |  |  |  |
| FLT3 | 55 |  |  |  |  |  |  |  |  |  |  |  |  |  |  |  |  |
| FGF7 | 54 |  |  |  |  |  |  |  |  |  |  |  |  |  |  |  |  |
| JAK3 | 54 |  |  |  |  |  |  |  |  |  |  |  |  |  |  |  |  |
| LAMA1 | 53 |  |  |  |  |  |  |  |  |  |  |  |  |  |  |  |  |
| COL1A2 | 52 |  |  | ITGA2 | 51 |  |  | FGF16 | 16 |  |  | KIT | 10 |  |  | VEGFB | 4 |
| PDGFA | 52 |  |  |  |  |  |  |  |  |  |  |  |  |  |  |  |  |
| CSF1R | 51 |  |  | LAMA5 | 36 |  |  |  |  |  |  | STK11 | 12 |  |  |  |  |
| EPHA2 | 51 |  |  |  |  |  |  |  |  |  |  |  |  |  |  |  |  |
| MCL1 | 51 |  |  |  |  |  |  |  |  |  |  |  |  |  |  |  |  |
| NTRK1 | 51 |  |  |  |  |  |  |  |  |  |  |  |  |  |  |  |  |
| COL4A2 | 50 |  |  | ITGA4 | 46 |  |  | CREB1 | 17 |  |  | LPAR3 | 20 |  |  | YWHAG | 9 |
| FGF4 | 50 |  |  |  |  |  |  |  |  |  |  |  |  |  |  |  |  |
| LAMC2 | 50 |  |  |  |  |  |  |  |  |  |  |  |  |  |  |  |  |
| NFKB1 | 50 |  |  |  |  |  |  |  |  |  |  |  |  |  |  |  |  |
| RPTOR | 50 |  |  |  |  |  |  |  |  |  |  |  |  |  |  |  |  |
| BCL2L11 | 49 |  |  | COL6A3 | 39 |  |  | MET | 23 |  |  | FGFR4 | 7 |  |  | PHLPP1 | 3 |
| COL4A1 | 49 |  |  | ITGA3 | 48 |  |  | ERBB3 | 27 |  |  | LPAR2 | 20 |  |  | YWHAE | 8 |
| CSF1 | 49 |  |  | LAMA4 | 37 |  |  |  |  |  |  | SGK1 | 10 |  |  |  |  |
| FGFR4 | 49 |  |  |  |  |  |  |  |  |  |  |  |  |  |  |  |  |
| FIGF | 49 |  |  |  |  |  |  |  |  |  |  |  |  |  |  |  |  |
| NTRK2 | 49 |  |  |  |  |  |  |  |  |  |  |  |  |  |  |  |  |
| CASP9 | 48 |  |  | COL6A6 | 34 |  |  | MCL1 | 18 |  |  | FLT1 | 7 |  |  | PPP2CB | 15 |
| FGF9 | 47 |  |  |  |  |  |  |  |  |  |  |  |  |  |  |  |  |
| LAMC1 | 47 |  |  |  |  |  |  |  |  |  |  |  |  |  |  |  |  |
| CDKN1A | 46 |  |  | HGF | 30 |  |  | FGF9 | 20 |  |  | GRB2 | 21 |  |  | RAC1 | 5 |
| COL4A3 | 46 |  |  | ITGA5 | 49 |  |  | COMP | 2 |  |  | LPAR4 | 18 |  |  | YWHAH | 10 |
| MAP2K2 | 46 |  |  |  |  |  |  |  |  |  |  |  |  |  |  |  |  |
| COL6A2 | 45 |  |  | ITGA9 | 46 |  |  | BDNF | 27 |  |  | MAPK1 | 31 |  |  |  |  |
| COL6A3 | 45 |  |  | ITGAV | 51 |  |  | BCL2L11 | 17 |  |  | NFKB1 | 9 |  |  |  |  |
| EIF4E | 45 |  |  | SOS1 | 19 |  |  |  |  |  |  |  |  |  |  |  |  |
| FGF22 | 45 |  |  |  |  |  |  |  |  |  |  |  |  |  |  |  |  |
| FGF3 | 45 |  |  |  |  |  |  |  |  |  |  |  |  |  |  |  |  |
| NTF3 | 45 |  |  |  |  |  |  |  |  |  |  |  |  |  |  |  |  |
| STK11 | 45 |  |  |  |  |  |  |  |  |  |  |  |  |  |  |  |  |
| FGF19 | 44 |  |  |  |  |  |  |  |  |  |  |  |  |  |  |  |  |
| LAMA3 | 44 |  |  |  |  |  |  |  |  |  |  |  |  |  |  |  |  |
| COL2A1 | 43 |  |  | ITGA2B | 49 |  |  | ERBB4 | 21 |  |  | LPAR1 | 21 |  |  | YWHAB | 14 |
| FGF16 | 43 |  |  |  |  |  |  |  |  |  |  |  |  |  |  |  |  |
| IL2RA | 43 |  |  |  |  |  |  |  |  |  |  |  |  |  |  |  |  |
| LAMA4 | 43 |  |  |  |  |  |  |  |  |  |  |  |  |  |  |  |  |
| YWHAQ | 43 |  |  |  |  |  |  |  |  |  |  |  |  |  |  |  |  |
| CDK4 | 42 |  |  | FGF4 | 14 |  |  | FGFR2 | 20 |  |  | GNG4 | 20 |  |  | PPP2R5C | 12 |
| COL4A4 | 42 |  |  | ITGA6 | 53 |  |  | CDKN1A | 17 |  |  | LPAR5 | 18 |  |  | YWHAQ | 9 |
| FGF18 | 42 |  |  |  |  |  |  |  |  |  |  |  |  |  |  |  |  |
| YWHAE | 42 |  |  |  |  |  |  |  |  |  |  |  |  |  |  |  |  |
| CD19 | 41 |  |  | ERBB2 | 22 |  |  | IGF1R | 30 |  |  | GNG10 | 20 |  |  | PPP2R3A | 11 |
| COL4A5 | 41 |  |  | ITGA7 | 45 |  |  | CCND1 | 30 |  |  | LPAR6 | 18 |  |  |  |  |
| GNB1 | 41 |  |  |  |  |  |  |  |  |  |  |  |  |  |  |  |  |
| LAMB2 | 41 |  |  |  |  |  |  |  |  |  |  |  |  |  |  |  |  |
| PPP2CB | 41 |  |  |  |  |  |  |  |  |  |  |  |  |  |  |  |  |
| RHEB | 41 |  |  |  |  |  |  |  |  |  |  |  |  |  |  |  |  |
| THBS2 | 41 |  |  |  |  |  |  |  |  |  |  |  |  |  |  |  |  |
| TSC1 | 41 |  |  |  |  |  |  |  |  |  |  |  |  |  |  |  |  |
| VEGFB | 41 |  |  |  |  |  |  |  |  |  |  |  |  |  |  |  |  |
| YWHAG | 41 |  |  |  |  |  |  |  |  |  |  |  |  |  |  |  |  |
| IL2RB | 40 |  |  |  |  |  |  |  |  |  |  |  |  |  |  |  |  |
| LAMA2 | 40 |  |  |  |  |  |  |  |  |  |  |  |  |  |  |  |  |
| LAMA5 | 40 |  |  |  |  |  |  |  |  |  |  |  |  |  |  |  |  |
| NOS3 | 40 |  |  |  |  |  |  |  |  |  |  |  |  |  |  |  |  |
| PRKAA1 | 40 |  |  |  |  |  |  |  |  |  |  |  |  |  |  |  |  |
| SYK | 40 |  |  |  |  |  |  |  |  |  |  |  |  |  |  |  |  |
| YWHAH | 40 |  |  |  |  |  |  |  |  |  |  |  |  |  |  |  |  |
| CDK2 | 38 |  |  | FGF2 | 26 |  |  | FGFR3 | 20 |  |  | GNG2 | 20 |  |  | PPP2R5B | 12 |
| COL4A6 | 38 |  |  | ITGA8 | 45 |  |  | CASP9 | 18 |  |  | MAP2K2 | 11 |  |  |  |  |
| COL9A2 | 38 |  |  | ITGB5 | 46 |  |  | AKT1 | 34 |  |  | NTRK2 | 9 |  |  |  |  |
| GNB3 | 38 |  |  |  |  |  |  |  |  |  |  |  |  |  |  |  |  |
| COL9A1 | 37 |  |  | ITGB4 | 51 |  |  | AKT2 | 17 |  |  | NTRK1 | 9 |  |  |  |  |
| EFNA5 | 37 |  |  | NGF | 14 |  |  |  |  |  |  |  |  |  |  |  |  |
| COL6A5 | 36 |  |  | ITGB1 | 57 |  |  | BCL2L1 | 21 |  |  | NGFR | 14 |  |  |  |  |
| COL6A6 | 36 |  |  | ITGB3 | 55 |  |  | ANGPT1 | 18 |  |  | NTF3 | 11 |  |  |  |  |
| GNB4 | 36 |  |  |  |  |  |  |  |  |  |  |  |  |  |  |  |  |
| PPP2R1B | 36 |  |  |  |  |  |  |  |  |  |  |  |  |  |  |  |  |
| PRKAA2 | 36 |  |  |  |  |  |  |  |  |  |  |  |  |  |  |  |  |
| SGK1 | 36 |  |  |  |  |  |  |  |  |  |  |  |  |  |  |  |  |
| TGFA | 36 |  |  |  |  |  |  |  |  |  |  |  |  |  |  |  |  |
| BCL2 | 35 |  |  | COL4A6 | 36 |  |  | PDGFB | 17 |  |  | EPHA2 | 6 |  |  | PDGFC | 4 |
| CCND2 | 35 |  |  | COL9A2 | 35 |  |  | KITLG | 20 |  |  | GNB3 | 21 |  |  | PPP2R2B | 18 |
| CHRM1 | 35 |  |  | ITGA1 | 52 |  |  | FGF22 | 18 |  |  | INSR | 11 |  |  | SGK3 | 3 |
| GNG12 | 35 |  |  |  |  |  |  |  |  |  |  |  |  |  |  |  |  |
| GNG2 | 35 |  |  |  |  |  |  |  |  |  |  |  |  |  |  |  |  |
| MLST8 | 35 |  |  |  |  |  |  |  |  |  |  |  |  |  |  |  |  |
| PIK3R6 | 35 |  |  |  |  |  |  |  |  |  |  |  |  |  |  |  |  |
| TLR2 | 35 |  |  |  |  |  |  |  |  |  |  |  |  |  |  |  |  |
| CCNE1 | 34 |  |  | EGFR | 30 |  |  | IGF2 | 22 |  |  | GNB5 | 20 |  |  | PPP2R2D | 11 |
| PDGFC | 34 |  |  |  |  |  |  |  |  |  |  |  |  |  |  |  |  |
| PPP2R2A | 34 |  |  |  |  |  |  |  |  |  |  |  |  |  |  |  |  |
| PRL | 34 |  |  |  |  |  |  |  |  |  |  |  |  |  |  |  |  |
| ATF4 | 33 |  |  | COL4A3 | 41 |  |  | PIK3CB | 25 |  |  | CHRM1 | 19 |  |  | FASLG | 2 |
| IL7R | 33 |  |  |  |  |  |  |  |  |  |  |  |  |  |  |  |  |
| LAMC3 | 33 |  |  |  |  |  |  |  |  |  |  |  |  |  |  |  |  |
| RELN | 33 |  |  |  |  |  |  |  |  |  |  |  |  |  |  |  |  |
| VTN | 33 |  |  |  |  |  |  |  |  |  |  |  |  |  |  |  |  |
| EFNA3 | 32 |  |  | LAMC2 | 37 |  |  |  |  |  |  |  |  |  |  |  |  |
| EFNA4 | 32 |  |  | LAMC3 | 30 |  |  |  |  |  |  |  |  |  |  |  |  |
| F2R | 32 |  |  |  |  |  |  |  |  |  |  |  |  |  |  |  |  |
| FASLG | 32 |  |  |  |  |  |  |  |  |  |  |  |  |  |  |  |  |
| FLT3LG | 32 |  |  |  |  |  |  |  |  |  |  |  |  |  |  |  |  |
| GNB5 | 32 |  |  |  |  |  |  |  |  |  |  |  |  |  |  |  |  |
| GNG10 | 32 |  |  |  |  |  |  |  |  |  |  |  |  |  |  |  |  |
| GNG4 | 32 |  |  |  |  |  |  |  |  |  |  |  |  |  |  |  |  |
| GNGT2 | 32 |  |  |  |  |  |  |  |  |  |  |  |  |  |  |  |  |
| IKBKB | 32 |  |  |  |  |  |  |  |  |  |  |  |  |  |  |  |  |
| IL6R | 31 |  |  |  |  |  |  |  |  |  |  |  |  |  |  |  |  |
| PHLPP2 | 31 |  |  |  |  |  |  |  |  |  |  |  |  |  |  |  |  |
| PPP2R2B | 31 |  |  |  |  |  |  |  |  |  |  |  |  |  |  |  |  |
| RPS6KB2 | 31 |  |  |  |  |  |  |  |  |  |  |  |  |  |  |  |  |
| COMP | 30 |  |  | ITGB6 | 46 |  |  |  |  |  |  | PDGFA | 11 |  |  |  |  |
| TNC | 30 |  |  |  |  |  |  |  |  |  |  |  |  |  |  |  |  |
| LPAR1 | 29 |  |  |  |  |  |  |  |  |  |  |  |  |  |  |  |  |
| PHLPP1 | 29 |  |  |  |  |  |  |  |  |  |  |  |  |  |  |  |  |
| PPP2R5C | 29 |  |  |  |  |  |  |  |  |  |  |  |  |  |  |  |  |
| PPP2R5D | 29 |  |  |  |  |  |  |  |  |  |  |  |  |  |  |  |  |
| CDK6 | 28 |  |  | FGF7 | 18 |  |  | FGFR1 | 20 |  |  | GNGT2 | 20 |  |  | PPP2R5E | 12 |
| IL4R | 28 |  |  |  |  |  |  |  |  |  |  |  |  |  |  |  |  |
| GHR | 27 |  |  |  |  |  |  |  |  |  |  |  |  |  |  |  |  |
| HSP90B1 | 27 |  |  |  |  |  |  |  |  |  |  |  |  |  |  |  |  |
| LPAR2 | 27 |  |  |  |  |  |  |  |  |  |  |  |  |  |  |  |  |
| LPAR3 | 27 |  |  |  |  |  |  |  |  |  |  |  |  |  |  |  |  |
| PPP2R5E | 27 |  |  |  |  |  |  |  |  |  |  |  |  |  |  |  |  |
| GH1 | 26 |  |  |  |  |  |  |  |  |  |  |  |  |  |  |  |  |
| PPP2R5A | 26 |  |  |  |  |  |  |  |  |  |  |  |  |  |  |  |  |
| PPP2R5B | 26 |  |  |  |  |  |  |  |  |  |  |  |  |  |  |  |  |
| THBS3 | 26 |  |  |  |  |  |  |  |  |  |  |  |  |  |  |  |  |
| TNXB | 26 |  |  |  |  |  |  |  |  |  |  |  |  |  |  |  |  |
| CSH1 | 25 |  |  | LAMB1 | 43 |  |  |  |  |  |  | TP53 | 26 |  |  |  |  |
| TNN | 25 |  |  |  |  |  |  |  |  |  |  |  |  |  |  |  |  |
| CCND3 | 24 |  |  | EGF | 27 |  |  | JAK1 | 18 |  |  | GNB4 | 21 |  |  | PPP2R2C | 11 |
| CHAD | 24 |  |  | INS | 22 |  |  | FGF3 | 17 |  |  | GSK3B | 19 |  |  | RPTOR | 9 |
| SOS2 | 24 |  |  |  |  |  |  |  |  |  |  |  |  |  |  |  |  |
| BAD | 23 |  |  | COL4A5 | 39 |  |  | PDGFRA | 20 |  |  | EIF4E | 13 |  |  | FN1 | 8 |
| CRTC2 | 23 |  |  | LAMA3 | 36 |  |  |  |  |  |  | RPS6KB2 | 9 |  |  |  |  |
| DDIT4 | 23 |  |  | LAMC1 | 39 |  |  |  |  |  |  | TSC2 | 15 |  |  |  |  |
| PRLR | 23 |  |  |  |  |  |  |  |  |  |  |  |  |  |  |  |  |
| SGK3 | 23 |  |  |  |  |  |  |  |  |  |  |  |  |  |  |  |  |
| CDC37 | 22 |  |  | FGF1 | 15 |  |  | FOXO3 | 19 |  |  | GNG12 | 20 |  |  | PPP2R5A | 12 |
| CHRM2 | 21 |  |  | ITGA10 | 46 |  |  | FGF19 | 18 |  |  | IRS1 | 24 |  |  | TGFA | 3 |
| CSH2 | 21 |  |  | LAMB2 | 34 |  |  |  |  |  |  | TSC1 | 13 |  |  |  |  |
| LPAR4 | 21 |  |  |  |  |  |  |  |  |  |  |  |  |  |  |  |  |
| LPAR5 | 21 |  |  |  |  |  |  |  |  |  |  |  |  |  |  |  |  |
| LPAR6 | 21 |  |  |  |  |  |  |  |  |  |  |  |  |  |  |  |  |
| TNR | 21 |  |  |  |  |  |  |  |  |  |  |  |  |  |  |  |  |
| IFNA10 | 20 |  |  |  |  |  |  |  |  |  |  |  |  |  |  |  |  |
| IFNA16 | 20 |  |  |  |  |  |  |  |  |  |  |  |  |  |  |  |  |
| IFNA17 | 20 |  |  |  |  |  |  |  |  |  |  |  |  |  |  |  |  |
| IFNA21 | 20 |  |  |  |  |  |  |  |  |  |  |  |  |  |  |  |  |
| IFNA4 | 20 |  |  |  |  |  |  |  |  |  |  |  |  |  |  |  |  |
| MYB | 20 |  |  |  |  |  |  |  |  |  |  |  |  |  |  |  |  |
| PPP2R2C | 20 |  |  |  |  |  |  |  |  |  |  |  |  |  |  |  |  |
| PPP2R2D | 20 |  |  |  |  |  |  |  |  |  |  |  |  |  |  |  |  |
| OSMR | 18 |  |  |  |  |  |  |  |  |  |  |  |  |  |  |  |  |
| SGK2 | 18 |  |  |  |  |  |  |  |  |  |  |  |  |  |  |  |  |
| ATF2 | 17 |  |  | COL4A2 | 46 |  |  | PTEN | 30 |  |  | CDK6 | 5 |  |  | EPO | 3 |
| CREB5 | 17 |  |  | LAMA2 | 35 |  |  |  |  |  |  | RPS6KB1 | 15 |  |  |  |  |
| G6PC | 17 |  |  |  |  |  |  |  |  |  |  |  |  |  |  |  |  |
| PPP2R3A | 17 |  |  |  |  |  |  |  |  |  |  |  |  |  |  |  |  |
| EIF4B | 16 |  |  | PTK2 | 57 |  |  |  |  |  |  |  |  |  |  |  |  |
| IFNAR2 | 16 |  |  |  |  |  |  |  |  |  |  |  |  |  |  |  |  |
| RBL2 | 16 |  |  |  |  |  |  |  |  |  |  |  |  |  |  |  |  |
| CREB3 | 15 |  |  | KDR | 23 |  |  |  |  |  |  | RELA | 10 |  |  |  |  |
| CREB3L2 | 15 |  |  | KRAS | 18 |  |  |  |  |  |  | RELN | 3 |  |  |  |  |
| ATF6B | 14 |  |  | COL4A4 | 39 |  |  | PDGFRB | 24 |  |  | CHRM2 | 18 |  |  | FLT3 | 6 |
| CREB3L4 | 14 |  |  | LAMA1 | 40 |  |  |  |  |  |  | RHEB | 15 |  |  |  |  |
| GYS1 | 13 |  |  |  |  |  |  |  |  |  |  |  |  |  |  |  |  |
| PCK2 | 13 |  |  |  |  |  |  |  |  |  |  |  |  |  |  |  |  |
| RXRA | 13 |  |  |  |  |  |  |  |  |  |  |  |  |  |  |  |  |
| PIK3AP1 | 12 |  |  |  |  |  |  |  |  |  |  |  |  |  |  |  |  |
| GYS2 | 11 |  |  |  |  |  |  |  |  |  |  |  |  |  |  |  |  |
| GH2 | 9 |  |  |  |  |  |  |  |  |  |  |  |  |  |  |  |  |
| PKN1 | 8 |  |  |  |  |  |  |  |  |  |  |  |  |  |  |  |  |
| PKN2 | 8 |  |  |  |  |  |  |  |  |  |  |  |  |  |  |  |  |
| TCL1A | 8 |  |  |  |  |  |  |  |  |  |  |  |  |  |  |  |  |
| PKN3 | 6 |  |  |  |  |  |  |  |  |  |  |  |  |  |  |  |  |
| EIF4E1B | 5 |  |  | THBS1 | 40 |  |  |  |  |  |  |  |  |  |  |  |  |
| THEM4 | 5 |  |  |  |  |  |  |  |  |  |  |  |  |  |  |  |  |
| MAGI2 | 4 |  |  |  |  |  |  |  |  |  |  |  |  |  |  |  |  |
| MTCP1 | 4 |  |  |  |  |  |  |  |  |  |  |  |  |  |  |  |  |
| EIF4E2 | 2 |  |  | THBS2 | 32 |  |  |  |  |  |  |  |  |  |  |  |  |
| G6PC2 | 2 |  |  |  |  |  |  |  |  |  |  |  |  |  |  |  |  |
| MAGI1 | 2 |  |  |  |  |  |  |  |  |  |  |  |  |  |  |  |  |

| supplementary table8_mTOR pathway | | | | | | | | | | | | | | | | | |
| --- | --- | --- | --- | --- | --- | --- | --- | --- | --- | --- | --- | --- | --- | --- | --- | --- | --- |
| genesymbol | degree |  | module1 | Genesymbol | degree |  | module2 | Genesymbol | degree |  | module3 | Genesymbol | degree |  | module4 | Genesymbol | degree |
| MTOR | 71 |  |  |  |  |  |  |  |  |  |  |  |  |  |  |  |  |
| AKT1 | 67 |  |  | WNT7B | 26 |  |  | SGK1 | 16 |  |  | ATP6V1H | 8 |  |  | PTEN | 8 |
| RPTOR | 62 |  |  |  |  |  |  |  |  |  |  |  |  |  |  |  |  |
| INS | 58 |  |  |  |  |  |  |  |  |  |  |  |  |  |  |  |  |
| TSC2 | 58 |  |  |  |  |  |  |  |  |  |  |  |  |  |  |  |  |
| GSK3B | 57 |  |  |  |  |  |  |  |  |  |  |  |  |  |  |  |  |
| PTEN | 56 |  |  |  |  |  |  |  |  |  |  |  |  |  |  |  |  |
| RHEB | 56 |  |  |  |  |  |  |  |  |  |  |  |  |  |  |  |  |
| RPS6KB1 | 53 |  |  |  |  |  |  |  |  |  |  |  |  |  |  |  |  |
| RHOA | 49 |  |  |  |  |  |  |  |  |  |  |  |  |  |  |  |  |
| MAPK1 | 47 |  |  |  |  |  |  |  |  |  |  |  |  |  |  |  |  |
| IRS1 | 45 |  |  |  |  |  |  |  |  |  |  |  |  |  |  |  |  |
| KRAS | 45 |  |  |  |  |  |  |  |  |  |  |  |  |  |  |  |  |
| RICTOR | 45 |  |  |  |  |  |  |  |  |  |  |  |  |  |  |  |  |
| EIF4E | 44 |  |  | FZD4 | 26 |  |  | PIK3R1 | 11 |  |  |  |  |  |  |  |  |
| TSC1 | 44 |  |  |  |  |  |  |  |  |  |  |  |  |  |  |  |  |
| AKT1S1 | 42 |  |  | WNT10A | 26 |  |  | IGF1R | 19 |  |  | ATP6V1G2 | 8 |  |  | STK11 | 6 |
| MLST8 | 42 |  |  |  |  |  |  |  |  |  |  |  |  |  |  |  |  |
| PDPK1 | 41 |  |  |  |  |  |  |  |  |  |  |  |  |  |  |  |  |
| WNT1 | 40 |  |  |  |  |  |  |  |  |  |  |  |  |  |  |  |  |
| AKT2 | 39 |  |  | WNT9B | 26 |  |  | MAP2K1 | 14 |  |  | ATP6V1C2 | 8 |  |  | PDPK1 | 7 |
| AKT3 | 39 |  |  | WNT4 | 26 |  |  | MAPKAP1 | 14 |  |  | ATP6V1E1 | 8 |  |  | MTOR | 8 |
| PIK3CA | 39 |  |  |  |  |  |  |  |  |  |  |  |  |  |  |  |  |
| IGF1R | 38 |  |  |  |  |  |  |  |  |  |  |  |  |  |  |  |  |
| LAMTOR3 | 38 |  |  |  |  |  |  |  |  |  |  |  |  |  |  |  |  |
| INSR | 37 |  |  |  |  |  |  |  |  |  |  |  |  |  |  |  |  |
| WNT5A | 36 |  |  |  |  |  |  |  |  |  |  |  |  |  |  |  |  |
| LAMTOR1 | 35 |  |  |  |  |  |  |  |  |  |  |  |  |  |  |  |  |
| DVL2 | 34 |  |  | DVL3 | 26 |  |  | AKT2 | 21 |  |  |  |  |  |  |  |  |
| LAMTOR5 | 34 |  |  |  |  |  |  |  |  |  |  |  |  |  |  |  |  |
| WNT2 | 34 |  |  |  |  |  |  |  |  |  |  |  |  |  |  |  |  |
| WNT3A | 34 |  |  |  |  |  |  |  |  |  |  |  |  |  |  |  |  |
| SGK1 | 33 |  |  |  |  |  |  |  |  |  |  |  |  |  |  |  |  |
| FZD3 | 32 |  |  |  |  |  |  |  |  |  |  |  |  |  |  |  |  |
| RAF1 | 32 |  |  |  |  |  |  |  |  |  |  |  |  |  |  |  |  |
| RRAGC | 32 |  |  |  |  |  |  |  |  |  |  |  |  |  |  |  |  |
| TBC1D7 | 32 |  |  |  |  |  |  |  |  |  |  |  |  |  |  |  |  |
| GRB2 | 31 |  |  |  |  |  |  |  |  |  |  |  |  |  |  |  |  |
| LRP5 | 31 |  |  |  |  |  |  |  |  |  |  |  |  |  |  |  |  |
| MAP2K1 | 31 |  |  |  |  |  |  |  |  |  |  |  |  |  |  |  |  |
| PIK3CB | 31 |  |  |  |  |  |  |  |  |  |  |  |  |  |  |  |  |
| STK11 | 31 |  |  |  |  |  |  |  |  |  |  |  |  |  |  |  |  |
| WNT9A | 31 |  |  |  |  |  |  |  |  |  |  |  |  |  |  |  |  |
| WNT9B | 31 |  |  |  |  |  |  |  |  |  |  |  |  |  |  |  |  |
| DVL3 | 30 |  |  | WNT5A | 26 |  |  | KRAS | 17 |  |  |  |  |  |  |  |  |
| LRP6 | 30 |  |  |  |  |  |  |  |  |  |  |  |  |  |  |  |  |
| PIK3R1 | 30 |  |  |  |  |  |  |  |  |  |  |  |  |  |  |  |  |
| WNT10B | 30 |  |  |  |  |  |  |  |  |  |  |  |  |  |  |  |  |
| WNT3 | 30 |  |  |  |  |  |  |  |  |  |  |  |  |  |  |  |  |
| WNT4 | 30 |  |  |  |  |  |  |  |  |  |  |  |  |  |  |  |  |
| FZD1 | 29 |  |  | FZD2 | 26 |  |  |  |  |  |  |  |  |  |  |  |  |
| FZD4 | 29 |  |  |  |  |  |  |  |  |  |  |  |  |  |  |  |  |
| PRKAA1 | 29 |  |  |  |  |  |  |  |  |  |  |  |  |  |  |  |  |
| WNT2B | 29 |  |  |  |  |  |  |  |  |  |  |  |  |  |  |  |  |
| FZD2 | 28 |  |  |  |  |  |  |  |  |  |  |  |  |  |  |  |  |
| FZD5 | 28 |  |  |  |  |  |  |  |  |  |  |  |  |  |  |  |  |
| FZD7 | 28 |  |  |  |  |  |  |  |  |  |  |  |  |  |  |  |  |
| PRKAA2 | 28 |  |  |  |  |  |  |  |  |  |  |  |  |  |  |  |  |
| RPS6KB2 | 28 |  |  |  |  |  |  |  |  |  |  |  |  |  |  |  |  |
| WNT6 | 28 |  |  |  |  |  |  |  |  |  |  |  |  |  |  |  |  |
| WNT7B | 28 |  |  |  |  |  |  |  |  |  |  |  |  |  |  |  |  |
| WNT8A | 28 |  |  |  |  |  |  |  |  |  |  |  |  |  |  |  |  |
| WNT8B | 28 |  |  |  |  |  |  |  |  |  |  |  |  |  |  |  |  |
| FZD6 | 27 |  |  |  |  |  |  |  |  |  |  |  |  |  |  |  |  |
| FZD8 | 27 |  |  |  |  |  |  |  |  |  |  |  |  |  |  |  |  |
| MAPKAP1 | 27 |  |  |  |  |  |  |  |  |  |  |  |  |  |  |  |  |
| SESN2 | 27 |  |  |  |  |  |  |  |  |  |  |  |  |  |  |  |  |
| WNT10A | 27 |  |  |  |  |  |  |  |  |  |  |  |  |  |  |  |  |
| PRKCA | 26 |  |  |  |  |  |  |  |  |  |  |  |  |  |  |  |  |
| SOS1 | 26 |  |  |  |  |  |  |  |  |  |  |  |  |  |  |  |  |
| RPS6KA1 | 25 |  |  |  |  |  |  |  |  |  |  |  |  |  |  |  |  |
| RPS6KA2 | 25 |  |  |  |  |  |  |  |  |  |  |  |  |  |  |  |  |
| NPRL2 | 23 |  |  |  |  |  |  |  |  |  |  |  |  |  |  |  |  |
| PIK3CD | 23 |  |  |  |  |  |  |  |  |  |  |  |  |  |  |  |  |
| RPS6KA3 | 22 |  |  |  |  |  |  |  |  |  |  |  |  |  |  |  |  |
| BRAF | 21 |  |  | WNT2 | 26 |  |  | TSC1 | 18 |  |  |  |  |  |  |  |  |
| EIF4B | 21 |  |  | FZD5 | 26 |  |  | MAPK1 | 18 |  |  |  |  |  |  |  |  |
| PIK3R3 | 21 |  |  |  |  |  |  |  |  |  |  |  |  |  |  |  |  |
| PRKCB | 21 |  |  |  |  |  |  |  |  |  |  |  |  |  |  |  |  |
| DEPDC5 | 20 |  |  | WNT8B | 26 |  |  | EIF4E | 19 |  |  |  |  |  |  |  |  |
| FZD9 | 20 |  |  |  |  |  |  |  |  |  |  |  |  |  |  |  |  |
| PRR5 | 20 |  |  |  |  |  |  |  |  |  |  |  |  |  |  |  |  |
| ULK1 | 20 |  |  |  |  |  |  |  |  |  |  |  |  |  |  |  |  |
| DDIT4 | 19 |  |  | FZD1 | 26 |  |  | RPS6KB2 | 15 |  |  |  |  |  |  |  |  |
| MAP2K2 | 19 |  |  |  |  |  |  |  |  |  |  |  |  |  |  |  |  |
| WDR24 | 19 |  |  |  |  |  |  |  |  |  |  |  |  |  |  |  |  |
| FNIP1 | 18 |  |  | LRP6 | 26 |  |  |  |  |  |  |  |  |  |  |  |  |
| GRB10 | 18 |  |  |  |  |  |  |  |  |  |  |  |  |  |  |  |  |
| IKBKB | 18 |  |  |  |  |  |  |  |  |  |  |  |  |  |  |  |  |
| SEH1L | 18 |  |  |  |  |  |  |  |  |  |  |  |  |  |  |  |  |
| MIOS | 17 |  |  |  |  |  |  |  |  |  |  |  |  |  |  |  |  |
| WDR59 | 17 |  |  |  |  |  |  |  |  |  |  |  |  |  |  |  |  |
| GATSL3 | 16 |  |  |  |  |  |  |  |  |  |  |  |  |  |  |  |  |
| TNF | 16 |  |  |  |  |  |  |  |  |  |  |  |  |  |  |  |  |
| ATP6V1A | 13 |  |  | WNT8A | 26 |  |  | AKT1S1 | 17 |  |  | ATP6V1F | 8 |  |  | GRB2 | 7 |
| ATP6V1C1 | 13 |  |  | FZD6 | 26 |  |  | RICTOR | 20 |  |  | ATP6V1G3 | 8 |  |  | AKT1 | 8 |
| ATP6V1C2 | 13 |  |  | WNT10B | 26 |  |  | PIK3CB | 16 |  |  | ATP6V1C1 | 8 |  |  | PIK3CA | 8 |
| ATP6V1E1 | 13 |  |  | LRP5 | 26 |  |  | RPS6KB1 | 20 |  |  | ATP6V1E2 | 8 |  |  | IRS1 | 8 |
| ATP6V1E2 | 13 |  |  | FZD3 | 26 |  |  | MLST8 | 15 |  |  | ATP6V1A | 8 |  |  | RHOA | 8 |
| ATP6V1G2 | 13 |  |  | FZD8 | 26 |  |  | GSK3B | 17 |  |  |  |  |  |  |  |  |
| ATP6V1G3 | 13 |  |  | WNT3 | 26 |  |  | TSC2 | 21 |  |  |  |  |  |  |  |  |
| ATP6V1H | 13 |  |  | WNT9A | 26 |  |  | INS | 19 |  |  |  |  |  |  |  |  |
| GATSL2 | 13 |  |  |  |  |  |  |  |  |  |  |  |  |  |  |  |  |
| TTI1 | 12 |  |  |  |  |  |  |  |  |  |  |  |  |  |  |  |  |
| FNIP2 | 11 |  |  | WNT1 | 26 |  |  |  |  |  |  |  |  |  |  |  |  |
| SOS2 | 11 |  |  |  |  |  |  |  |  |  |  |  |  |  |  |  |  |
| ATP6V1F | 10 |  |  | WNT2B | 26 |  |  | AKT3 | 21 |  |  |  |  |  |  |  |  |
| PRKCG | 10 |  |  |  |  |  |  |  |  |  |  |  |  |  |  |  |  |
| ULK2 | 10 |  |  |  |  |  |  |  |  |  |  |  |  |  |  |  |  |
| RPS6KA6 | 9 |  |  |  |  |  |  |  |  |  |  |  |  |  |  |  |  |
| STRADA | 9 |  |  |  |  |  |  |  |  |  |  |  |  |  |  |  |  |
| TELO2 | 8 |  |  |  |  |  |  |  |  |  |  |  |  |  |  |  |  |
| CAB39 | 7 |  |  | WNT6 | 26 |  |  | RHEB | 18 |  |  |  |  |  |  |  |  |
| LPIN1 | 6 |  |  |  |  |  |  |  |  |  |  |  |  |  |  |  |  |
| EIF4E1B | 5 |  |  | WNT3A | 26 |  |  |  |  |  |  |  |  |  |  |  |  |
| SLC7A5 | 5 |  |  |  |  |  |  |  |  |  |  |  |  |  |  |  |  |
| STRADB | 5 |  |  |  |  |  |  |  |  |  |  |  |  |  |  |  |  |
| CLIP1 | 3 |  |  | DVL2 | 26 |  |  | RPTOR | 20 |  |  |  |  |  |  |  |  |
| SLC3A2 | 3 |  |  |  |  |  |  |  |  |  |  |  |  |  |  |  |  |
| EIF4E2 | 2 |  |  | FZD7 | 26 |  |  |  |  |  |  |  |  |  |  |  |  |
| RNF152 | 2 |  |  |  |  |  |  |  |  |  |  |  |  |  |  |  |  |
